# A unique role of p53 haploinsufficiency or loss in the development of acute myeloid leukemia with FLT3-ITD mutation

**DOI:** 10.1038/s41375-021-01452-6

**Published:** 2021-11-03

**Authors:** Min Yang, Zengkai Pan, Kezhi Huang, Guntram Büsche, Hongyun Liu, Gudrun Göhring, Regina Rumpel, Oliver Dittrich-Breiholz, Steven Talbot, Michaela Scherr, Anuhar Chaturvedi, Matthias Eder, Julia Skokowa, Jianfeng Zhou, Karl Welte, Nils von Neuhoff, Ligen Liu, Arnold Ganser, Zhixiong Li

**Affiliations:** 1 Department of Hematology, Hemostasis, Oncology, and Stem Cell Transplantation, Hannover Medical School, Hannover, Germany; 2 Institute of Pathology, Hannover Medical School, Hannover, Germany; 3 Department of Human Genetics, Hannover Medical School, Hannover, Germany; 4 Institute for Laboratory Animal Science and Central Animal Facility, Hannover Medical School, Hannover, Germany; 5 Research Core Unit Genomics, Hannover Medical School, Hannover, Germany; 6 Department of Hematology, Oncology, Clinical Immunology, University of Tübingen, Tübingen, Germany; 7 Department of Hematology, Tongji Hospital, Tongji Medical College, Huazhong University of Science and Technology, Wuhan, China; 8 University Children’s Hospital, Department of General Pediatrics and Pediatric Hematology and Oncology, Tübingen, Germany; 9 AML Diagnostic Laboratory, Department of Pediatric Hematology-Oncology, University of Duisburg-Essen, Essen, Germany; 10 Department of Hematology, Shanghai Tongren Hospital, Shanghai Jiao Tong University School of Medicine, Shanghai, China; 11 Present Address: National Research Center for Translational Medicine, Ruijin Hospital, Shanghai Jiao Tong University School of Medicine, Shanghai, China; 12 Present Address: Guangdong Provincial Key Laboratory of Malignant Tumor Epigenetics and Gene Regulation, and Department of Hematology, Sun Yat-Sen Memorial Hospital, Sun Yat-Sen University, Guangzhou, China

**Keywords:** Cancer models, Preclinical research

## Abstract

With an incidence of ~50%, the absence or reduced protein level of p53 is much more common than *TP53* mutations in acute myeloid leukemia (AML). AML with *FLT3*-ITD (internal tandem duplication) mutations has an unfavorable prognosis and is highly associated with wt-p53 dysfunction. While *TP53* mutation in the presence of FLT3-ITD does not induce AML in mice, it is not clear whether p53 haploinsufficiency or loss cooperates with *FLT3*-ITD in the induction of AML. Here, we generated *FLT3*-ITD knock-in; *p53* knockout (heterozygous and homozygous) double-transgenic mice and found that both alterations strongly cooperated in the induction of cytogenetically normal AML without increasing the self-renewal potential. At the molecular level, we found the strong upregulation of *Htra3* and the downregulation of *Lin28a*, leading to enhanced proliferation and the inhibition of apoptosis and differentiation. The co-occurrence of *Htra3* overexpression and *Lin28a* knockdown, in the presence of FLT3-ITD, induced AML with similar morphology as leukemic cells from double-transgenic mice. These leukemic cells were highly sensitive to the proteasome inhibitor carfilzomib. Carfilzomib strongly enhanced the activity of targeting *AXL* (upstream of *FLT3*) against murine and human leukemic cells. Our results unravel a unique role of p53 haploinsufficiency or loss in the development of *FLT3*-ITD + AML.

## Introduction

Mutations in the *TP53* gene are very common in solid tumor with an incidence of up to 90% in certain cancers [1, 2]. *TP53* mutations are found in ~60% of AML patients with a complex karyotype but are rare in other AML patients (generally < 10%) [3]. Although *TP53* mutation appears to be an early event in the pathogenesis of AML [4], however, the co-occurrence of *TP53* mutations and *FLT3-*ITD is rare in AML [5]. In a mouse model, a p53 mutant in the presence of FLT3-ITD did not induce AML and did not shorten the survival of mice [6]. With an incidence of ~50%, absent/reduced protein level of p53 is much more common than *TP53* mutations in AML [3, 7–9]. Moreover, functional nuclear p53 protein has been shown to be strongly reduced in AML patients (up to 98% in patients with *FLT3*-ITD and *NPM1*mut) [10]. Deletion of 17p, to which *TP53* is mapped, occurs in AML as a single aberration or with additional chromosomal abnormalities, demonstrating an unfavorable cytogenetic category. AML with *FLT3*-ITD mutations is highly associated with wtp53 dysfunction via additional different ways such as *MDM2/MDM4* overexpression [11] (Supplementary Fig. 1), *SIRT1* overexpression (resulting p53 deacetylation), PI3K/AKT pathway activation (promoting MDM2-mediated p53 degradation), STAT/MAPK pathway activation with BCL2 accumulation (opposing p53 activity), disturbing the nucleocytoplasmic shuttling of p53 [10], dysregulation of NPM (in patients with *FLT3*-ITD and *NPM1* mutant) [12], and aberrant expression of certain miRNAs (e.g., miR-125b) [13]. Gene set enrichment analysis (GSEA) revealed that the transcriptome data of *FLT3*-mut AML patients are negatively associated with p53 and p63 targets (Supplementary Fig. 1). In an early mouse study, mutated/activated of F*LT3* by insertional mutagenesis was preferentially detected in *p53* and *p19*^*ARF−/−*^ knockout but not in wild-type tumors, suggesting that *FLT3* mutations may be particularly oncogenic in the absence of a functional p19^ARF^-MDM2-p53 pathway [14]. Activating mutations in *FLT3* have been identified in ~30–40% of patients with AML and are associated with an unfavorable prognosis [15, 16]. The recently published multi-institutional RATIFY study (including our institute) demonstrated a statistically significant improvement in the overall survival for *FLT3* + AML patients treated with midostaurin (targeting *FLT3*) and chemotherapy [15]. However, a beneficial effect of midostaurin appeared to be most pronounced in the *NPM1*^wt^/*FLT3*-ITD^low^ group. *FLT3*-ITD is a late event in the pathogenesis of AML. The identification of early cooperating events for *FLT3* mutations may improve our understanding of the pathogenesis of AML and lead to more efficient treatment and an improved outcome for AML patients.

In this study, we investigated whether p53 haploinsufficiency or loss cooperates with FLT3-ITD in the induction of AML. We found that both alterations strongly cooperated in the induction of cytogenetically normal AML without increasing the self-renewal potential. At the molecular level, strong upregulation of *Htra3* and downregulation of *Lin28a* were observed, that led to increased proliferation and the inhibition of apoptosis and differentiation. Overexpressed *Htra3* and knockdown of *Lin28a*, combined with the presence of *FLT3*-ITD, induced AML with similar morphology as leukemic cells from double-transgenic mice. Moreover, our data indicate that combined therapy of the proteasome inhibitor carfilzomib and targeting *AXL* (upstream of *FLT3*) might be a promising treatment option particularly in *FLT3*-ITD positive AML.

## Materials and methods

### *FLT3*-ITD knock-in and *p53* knockout mice, and generation of double-mutant mice

We obtained *FLT3*-ITD knock-in mice from the Jackson Laboratory (Bar Harbor, ME). *FLT3*-ITD knock-in mice with C57BL/6J background that carry the human internal tandem duplication (ITD) mutation W51 in exon 14 of the endogenous murine *Flt3* locus were generated in Gilliland’s laboratory [17]. The mice were backcrossed to C57BL/6 mice for 9 generations and were crossed to C57BL/6J at least once upon arrival at the Jackson Laboratory. Upon arrival at our animal facility, *FLT3*-ITD knock-in mice were crossed with C57BL/6J wild-type (WT) mice for at least four generations before crossing with *p53* knock-out (KO) mice. We confirmed the ITD mutation by sequencing. *FLT3*-ITD knock-in mice mainly developed CMML-like disease as reported (Supplementary Figs. 2 and 3). *p53* KO mice were obtained from the Jackson Laboratory. Exons 2–6 of the *p53* gene in the *p53* KO mice were replaced by a neomycin cassette in Weinberg’s laboratory [18]. Upon arrival at the Jackson Laboratory, the C57BL/6J strain was produced by backcrossing the *p53* KO mice at least five times to C57BL/6J inbred mice. Upon arrival at our animal facility, we crossed *p53* KO mice with C57BL/6J wild-type mice for at least two generations. FLT3-ITD knock-in mice were then crossed with *p53* KO mice to generate ITD/ITD; *p53*^+/−^ mice. ITD/ITD; *p53*^+/−^ mice were bred with each other to generate ITD/ITD; *p53*^+/−^, ITD/ITD; *p53*^−/−^, and ITD/ITD; *p53*^+/+^ (*p53* WT) mice. ITD/ITD; *p53* WT, ITD/ITD; *p53*^+/−^ and ITD/ITD; *p53*^−/−^ were born at ~22%, 65 and 13% frequencies, respectively. Few ITD/ITD and ITD/ITD; *p53*^+/−^ animals were obtained from breeding ITD/ITD; *p53*^+/−^ × ITD/ITD; *p53*^+/+^.

### Mouse monitoring and tumor phenotyping

All animals were kept in the animal laboratories of Hannover Medical School. Animal experiments were approved by the local ethical committee LAVES (Niedersächsisches Landesamt für Verbraucherschutz und Lebensmittelsicherheit) and performed according to their guidelines. Animals were killed for necropsy when termination criteria were met or the endpoints of the experiment were reached, or were analyzed when found dead before the onset of autolysis [19]. Tumor phenotyping were performed as described previously [19].

### RNA-sequencing and raw data processing and TaqMan assay

RNA-Sequencing and raw data processing were performed as previously described [20]. Quantitative (TaqMan) RT-PCR and GSEA were carried out as previously reported [21]. TaqMan probes were purchased from Applied Biosystems (Foster City, CA).

### Data analysis and statistics

FACS data were analyzed by FlowJo software (Tree Star, OR, USA). Statistical analyses and figures were generated by Graphpad Prism 6 or 7 (San Diego, CA). To compare difference of data in more than 3 groups, one-way ANOVA test was used. For data in 2 groups, Student’s t-test was used. The results were represented as the means ± SD. Significant differences in Kaplan–Meier survival were evaluated by the log-rank test. IC50 (half maximal inhibitory concentration) values were calculated by dose-response curves using nonlinear regression analysis (curve fit) after log-transformation. *P* values less than 0.05 were considered statistically significant.

### Multicolor fluorescence in situ hybridization (mFISH), colony-forming unit-spleen (CFU-S) assay, small-molecule inhibitor treatment, antibody arrays, and xenograft studies

Please refer to Supplementary Methods.

## Results

### Strong cooperating effect of FLT3-ITD and p53 haploinsufficiency or loss in the induction of AML

**Fig. 1:**
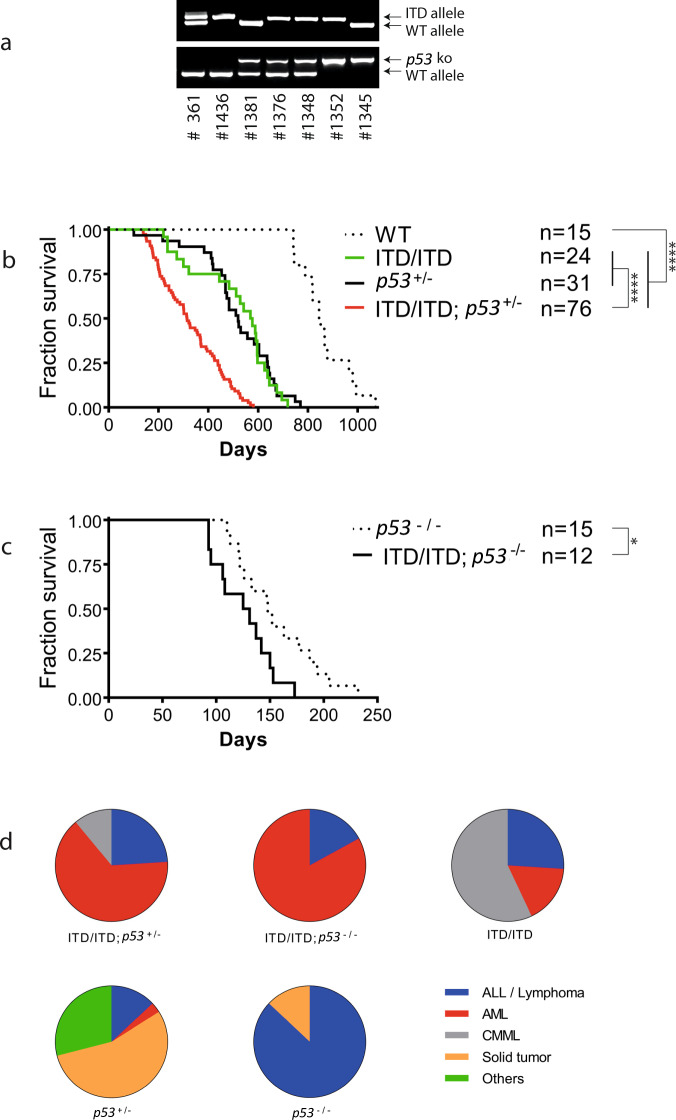
Dismal survival of mice with both *p53* knock-out and *FLT3*-ITD knock-in mutations. **a** Genotyping analyses of *p53* knockout and *FLT3*-ITD knock-in by PCR. The genotypes of offspring were detected by PCR amplification of *p53* or *FLT3*-ITD mutations in genomic DNA. Moreover, p53 haploinsufficient (p53^+/−^) mice (*n* = 3) showed a 1.8-fold (1.4–2.2-fold) reduction in *p53* mRNA expression quantitative PCR assay, while *p53* expression was completely absent in *p53*^−/−^ mice (*n* = 3) by the TaqMan assay. Mouse #361 = FLT3-ITD heterozygote, #1436 = ITD/ITD, #1381 = *p53*^+/−^, #1376 and #1348 = ITD/ITD; *p53*^+/−^, #1352 = ITD/ITD; *p53*^−/−^, #1345 = *p53*^−/−^. ITD/ITD = homozygous *FLT3*-ITD knock-in mice; *p53*^+/−^ = heterozygous *p53* knock-out mice; *p53*^−/−^ = homozygous *p53* knock-out mice; ITD/ITD; *p53*^+/−^ =mice with homozygous *FLT3*-ITD knock-in and heterozygous *p53* knock-out; ITD/ITD; *p53*^−/−^ = mice with homozygous *FLT3*-ITD knock-in and homozygous *p53* knock-out. **b** Survival curve of WT (wild-type), ITD/ITD; *p53*^+/−^, and ITD/ITD; *p53*^+/−^ mice. ***p < 0.001, ****p < 0.0001. **c** Survival curve of *p53*^−/−^ and ITD/ITD; *p53*^−/−^ mice. *p < 0.05. **d** Change in the disease spectrum by cooperation of *FLT3*-ITD and *p53* KO. All except three animals (not classifiable) from the survival curves (**b**, **c**) were included. Mice with biclonal disease (the coexistence of AML and ALL/lymphoma) were included in the AML group. In the *p53*^+/−^ group, one mouse developed both a solid tumor and lymphoma and was included in the solid tumor subgroup. One *p53*^−/−^ mouse developed mixed-phenotype acute leukemia and was included in the ALL/lymphoma subgroup. ALL /lymphoma = almost all are lymphoblastic lymphoma, others = histocytic sarcoma, adenoma/cystic diseases and few animals without pathological abnormality, although termination criteria were met.

**Fig. 2:**
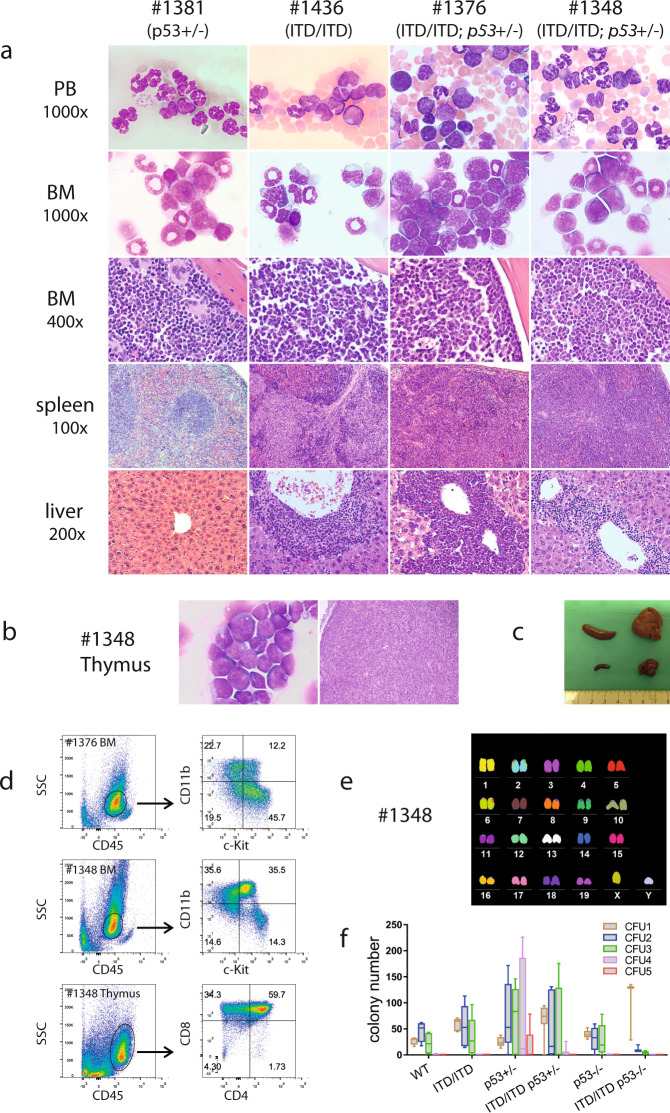
Development of cytogenetically normal AML induced by ITD/ITD and p53 haploinsufficiency or loss. **a** Representative Pappenheim-stained blood smears and bone marrow (BM) cytospins, and hematoxylin and eosin (H & E)-stained bone marrow, and liver from *p53*^+/−^, ITD/ITD, ITD/ITD; *p53*^+/−^ mice. Blood smear: there were no blasts and no increased monocytes in mouse #1381 (*p53*^+/−^). Monocytes were increased in mouse #1436 (ITD/ITD). Myeloblasts were strongly increased in mouse #1376 (ITD/ITD; *p53*^+/−^), while only few blasts were observed in mouse #1348 (ITD/ITD; *p53*^+/−^) with biclonal disease. BM cytospins: mouse #1381 showed a normal cellular component. Monocytes were strongly increased in mouse #1436. Myeloblats made up ~60 and 30% of all nucleated cells in mice #1376 and #1348, respectively. BM section: BM histology was consistent with the cellular constituents observed in cytospins. Note the presence of megakaryocytes in mouse #1381 and the reduction/absence of megakaryocytes in other animals with leukemia. Spleen section: white pulp and red pulp were present in mouse #1381, while the structures of the spleen were destroyed in the other 3 mice with leukemia, particularly in #1376 and #1348. Liver section: while mouse #1381 showed a normal liver structure, infiltration of leukemic cells was observed in the other 3 animals, particularly in #1376. Mouse #1381 developed adenocarcinoma and osteosarcoma (Supplementary Fig. 4). However, mouse #1381 demonstrated similar cytology and histology (PB, BM and spleen) as WT mice (data not shown), except for increased granulocytes in blood smear. Data from ITD/ITD; *p53*^−/−^ mice were presented in Supplementary Fig. 5. **b** Cytospin from mouse #1348 showing lymphoblastic cells in thymus (left panel). Histology showing strong infiltration of lymphoblastic cells in thymus from mouse #1348 (right panel). **c** Macroscopic characterization of ITD/ITD; *p53*^+/−^ mouse #1454. Note strong hepatosplenomegaly of mouse #1454 (upper panel) compared with a healthy control (lower panel). **d** Flow cytometric analysis of BM samples demonstrated a population of myeloblast/immature cells with lower side scatter (SSC) and CD45^dim^ expression in mice #1376 and #1348, which were positive for CD11b, c-Kit, and CD34 (Supplementary Fig. 6). Moreover, T-lymphoblastic lymphoma cells from the thymus of mouse #1348 showed lower SSC but high CD45 expression [42]. Tumor cells were positive for CD4, CD8 and CD3. Notably, there was no infiltration of T-ALL cells in the BM of mouse #1348. Thus, there were two separate clones present in mouse #1348 (AML in the BM, T-ALL in the thymus). **e** mFISH analysis of a representative *FLT3*-ITD; *p53*^+/−^ BM sample from mouse #1348 showing a normal karyotype (40, XY). Table S1 summarizes the karyotypes found in mice with AML (*n* = 12). **f** The replating colony assay demonstrated no increased self-renewal activity in vitro by double mutations. Cells from one ITD/ITD; *p53*^+/−^ mouse did not form colonies in the 1^st^ plating and cells from another 2 ITD/ITD; *p53*^+/−^ mice formed <6 colonies in the 2^nd^ replating, while cells from all animals from ITD/ITD and *p53*^+/−^ groups formed colonies in the 1^st^ plating and >14 colonies in the 2^nd^ replating (except for one mouse in the 2^nd^ replating). Consistent with early reports, *p53*^+/−^ increased self-renewal activity compared with WT *p53* (the only group in which many colonies continued to be observed after the 5th replating), which was abrogated in the presence of ITD/ITD. Results presented are the min to max of colony numbers from each replating. ITD/ITD, *n* = 5 mice; *p53*^+/−^, *n* = 5; ITD/ITD; *p53*^+/−^, *n* = 7; ITD/ITD; *p53*^−/−^, *n* = 3; *p53*^−/−^, *n* = 5; WT = 4.

To test whether FLT3-ITD and p53 haploinsufficiency or loss cooperate to initiate AML, we crossed *FLT3*-ITD knock-in mice [17] with *p53* knockout mice [18] to generate mice harboring both ITD/ITD (*FLT3*-ITD homozygous) and *p53* knockout mutations (Fig. 1a). Mice with both *FLT3*-ITD and *p53* knockout were viable and had no evident abnormalities at birth. ITD/ITD; *p53*^+/−^ (*FLT3*-ITD homozygous and *p53* heterozygous) mice survived much shorter than mice with ITD/ITD or *p53*^+/−^ alone (Fig. 1b). The median survivals of ITD/ITD; *p53*^+/−^ mice (*n* = 76), ITD/ITD mice (littermates/siblings of ITD/ITD; *p53*^+/−^ mice, *n* = 24), *p53*^+/−^ mice (*n* = 31), and WT mice (*n* = 15) were 315, 573, 520, and 845 days, respectively (*p* < 0.0001). Moreover, the median survival of ITD/ITD; *p53*^−/−^ mice (*FLT3*-ITD homozygous and *p53* homozygous, littermates/siblings of ITD/ITD; *p53*^+/−^ mice, *n* = 12) was further reduced (128 days) and significantly shorter than that of ITD/ITD; *p53*^+/−^ mice and p53^−/−^ mice (median survival: 148 days, *n* = 15) (Fig. 1c, *p* < 0.05). Importantly, the cooperation of both *FLT3*-ITD and *p53* KO also significantly altered the disease spectrum in mice (Fig. 1d). The vast majority of ITD/ITD; *p53*^+/−^ mice developed AML (65%) and acute lymphoblastic leukemia (ALL, 24%) (Figs. 1d and 2a, b), while AML was observed only in 17% of ITD/ITD mice (others developed CMML-like disease (57%, Fig. 2a) and acute lymphoblastic leukemia (ALL, 24%)), and *p53*^+/−^ mice mainly developed solid tumors (55%) and lymphoma (13%) as reported previously (Fig. 1d; Supplementary Fig. 4) [18]. The incidence of AML was further significantly increased in ITD/ITD; *p53*^−/−^ mice (83%) (Supplementary Figs. 5 and 6), while most *p53*^−/−^ mice developed lymphoma/lymphoblastic leukemia (87%) and none of the *p53*^−/−^ mice developed AML (Supplementary Fig. 7) as reported previously [18]. Compared with ITD/ITD; *p53* WT mice, the weight of the thymus in ITD/ITD; *p53*^+/−^ mice was significantly higher (mean: 869 mg vs. 506 mg), while there was no significant difference in terms of the blood content (WBC, HB, PLT), and the weights of the spleen and liver (Supplementary Fig. 8). Interestingly, 90% of ITD/ITD; *p53*^−/−^ and 52% of ITD/ITD; *p53*^+/−^ mice with acute leukemia demonstrated biclonal disease, with the coexistence of AML and ALL/lymphoma (e.g., #1348: Fig. 2a–d; #1352: Supplementary Fig. 5), while this phenomenon was observed only in one ITD/ITD mouse. Interestingly, similar to patients with Li-Fraumeni syndrome, two ITD/ITD; *p53*^+/−^ mice developed both osteosarcoma and AML (Supplementary Fig. 9). Importantly, AML and ALL cells from ITD/ITD; *p53*^+/−^ mice were transplantable (Supplementary Fig. 10). Of note, loss of heterozygosity (LOH) for *p53* was not observed in ITD/ITD; *p53*^+/−^ and *p53*^+/−^ mice. Taken together, our data indicate a strong cooperating effect of FLT3-ITD and p53 haploinsufficiency or loss in the induction of acute leukemia.

**Fig. 3:**
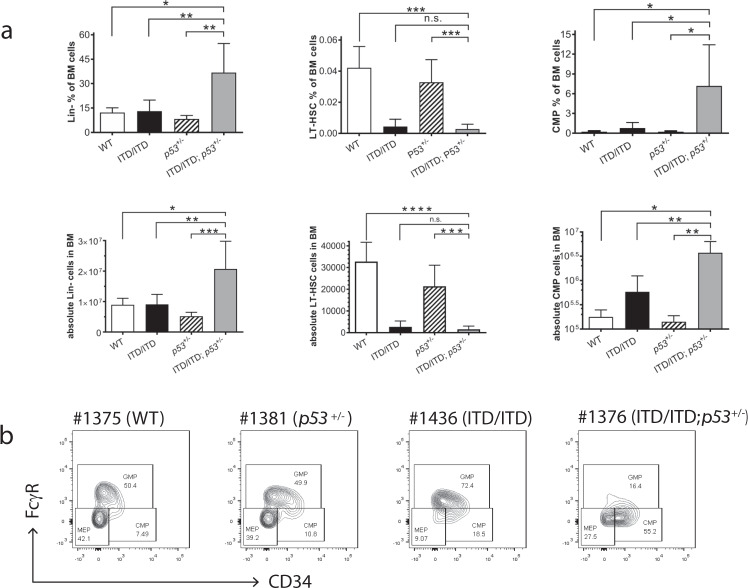
Hematopoietic stem/progenitor compartments in the BM of diseased mice. **a** Percentages and absolute cell number of Lin^−^, LT-HSC, and CMP cells in the BM. Data of other hematopoietic stem/progenitor cells were presented in Supplementary Fig. 9. Reduction of LT-HSCs in *FLT3*-ITD/ITD and ITD/ITD; *p53*^+/−^ mice is consistent with a recent published report demonstrating that *FLT3*-ITD knock-in impairs HSC quiescence/homeostasis, leading to the depletion of LT-HSC [43]. The data shown in graphs represent means ± SD. *p < 0.05, **p < 0.01, n.s. no significant difference. Lin^−^: lineage negative cells; LT-HSC: long-term hematopoietic stem cells (Lin^−^c-Kit^+^Sca-1^+^CD48^−^CD150^+^); CMP: common myeloid progenitors (Lin^−^IL-7Rα^−^c-Kit^+^Sca-1^−^CD34^+^FcgR^−^). WT (wild-type): *n* = 3 mice; ITD/ITD: *n* = 6; p53^+/−^: *n* = 6; ITD/ITD; p53^+/−^: *n* = 6. **b** Representative flow cytometric analysis showing increased leukemic CMP in mouse #1376. CMP was increased 66-fold in #1376 compared with WT mice. Of note, the frequencies of LSK, CMP and GMP in WT mice were very similar to published data (e.g., 0.29% LSK in our studies vs. 0.24% reported by Sitnicka et al. [44] and CMP: 0.23% vs. 0.13% [44]).

### Cooperation of *FLT3*-ITD and *p53* knockout induces cytogenetically normal AML without increased self-renewal potential

We performed multicolor fluorescence in situ hybridization (mFISH) analyses on the bone marrow, spleen or thymus cells from ITD/ITD; *p53*^+/−^ and ITD/ITD; *p53*^−/−^ mice. Unexpectedly, all analyzed murine leukemias (*n* = 12) showed normal karyotypes (Fig. 2e; Supplementary Fig. 9; Supplementary Table 1), indicating that p53 haploinsufficiency or loss per se does not lead to genetic instability, and genetic instability is not a major cause for AML and ALL development by the co-occurrence of *p53*KO and *FLT3*-ITD. Of note, a cooperation of genetic instability was likely needed for development of solid tumor induced by *p53*^+/−^ alone (Supplementary Fig. 4). Interestingly, p53 haploinsufficiency in the co-occurrence of ITD/ITD did not increase the self-renewal of hematopoietic stem/progenitor cells, as determined by the serial replating assays in vitro (Fig. 2f). The limiting dilution transplantation of leukemic cells in vivo demonstrated a 10-fold reduction in leukemic stem cell (LSC) frequency in ITD/ITD; *p53*^+/−^ leukemic cells compared with ITD/ITD cells (Supplementary Table 2). Moreover, the combination of ITD/ITD and p53 loss did not increase replating activity in vitro (Fig. 2f), suggesting that self-renewal activity did not increase. These findings suggest that AML development in our model is unlikely due to increased self-renewal of HSCs/HPCs, in contrast to that in *NPM1c/FLT3*-ITD and *FLT3*-ITD/*Dnmt3a* mutant mice [22, 23]. Recently, we showed that *FLT3*-N676K mutation has stronger transforming activity than *FLT3*-ITD, but leukemia induced by *FLT3*-N676K showed 100-fold less LSC frequency than *FLT3*-ITD [24]. Moreover, we observed a 10-fold lower LSC frequency by *FLT3*-ITD-TKD835 when assessed against *FLT3*-ITD (KH and ZL, unpublished data). These findings suggest that oncogenes/combinations with stronger leukemogenic potency may have a lower LSC frequency. Moreover, we found that Lin− cells were significantly increased in ITD/ITD; *p53*^+/−^ mice, and LT-HSCs were strongly reduced in *FLT3*-ITD/ITD and ITD/ITD; *p53*^+/−^ mice compared with that in WT and *p53*^+/−^ mice (Fig. 3a). Animals with only AML in the ITD/ITD; *p53*^+/−^ group showed a significant increase in common myeloid progenitors (CMPs) compared with that in ITD/ITD mice with CMML, *p53*^+/−^ and WT mice (Fig. 3a, b, Supplementary Fig. S9e), while granulocyte-monocyte progenitors (GMPs) in the ITD/ITD; *p53*^+/−^ group were not increased compared with that in the ITD/ITD-alone group or WT mice (Supplementary Fig. S9f). However, no difference was found regarding HSC, CMP and GMP in young non-diseased mice from all the groups (data not shown). This finding suggests that AML cells from the ITD/ITD; *p53*^+/−^ mice with only AML mirror CMPs and the block of differentiation from CMPs to GMPs might have contributed to the development of AML in our model.

### RNA sequencing reveals strong upregulation of *Htra3* and downregulation of *Lin28a*, leading to increased proliferation, the inhibition of differentiation and apoptosis, and leukemia development

**Fig. 4:**
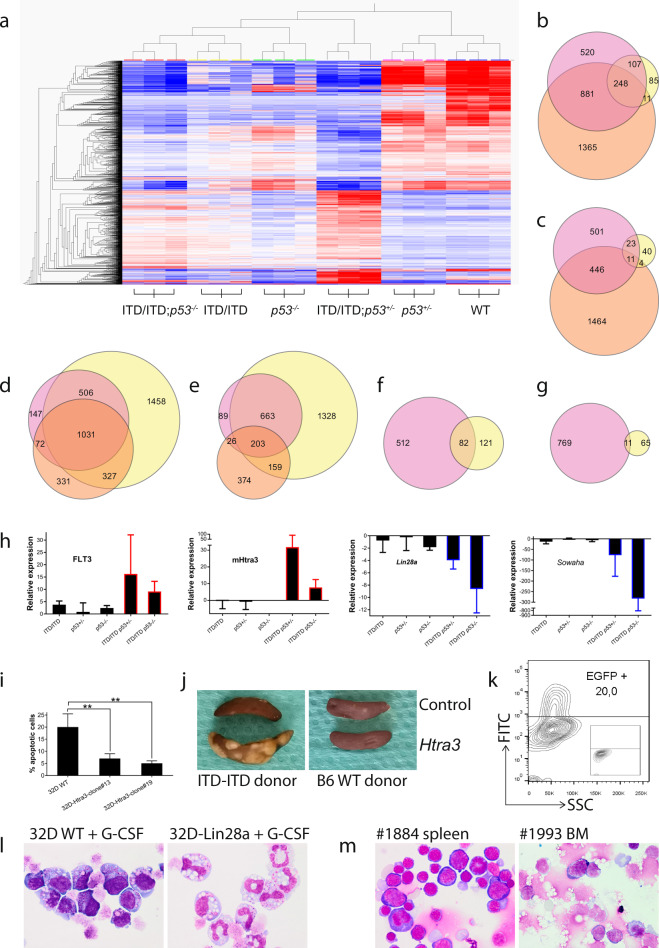
RNA-Sequencing analysis. **a** Normalized expression profiles of the top 2176 genes with the largest standard deviations across all samples. The dendrogram shows samples in all groups clustered as expected. RNA samples were taken from BM cells from WT, *p53*^+/−^, ITD/ITD; *p53*^+/−^, ITD/ITD; *p53*^−/−^, and *p53*^−/−^ mice (*n* = 3 for each group). There were only AML in BM in these ITD/ITD; *p53*^+/−^ (e.g., #1376, Fig. 2a) and ITD/ITD; *p53*^−/−^ mice, while *p53*^+/-^ (e.g., #1381, Fig. 2a) and *p53*^−/−^ mice did not show infiltration of tumor cells in BM. Differentially expressed p53 target genes in p53^+/−^ mice compared with ITD/ITD mice were presented in Supplementary Fig. 11. Venn diagrams show the overlap of differentially expressed genes among 3 data sets including ITD/ITD; *p53*^+/−^ (**b**: downregulated, **c**: upregulated). Pink = ITD/ITD vs WT; Yellow = *p53*^+/−^ vs WT; Orange = ITD/ITD; *p53*^+/−^ vs WT. Genes were considered to be differentially expressed based on DESeq2 statistics applying a corrected *p* value threshold (false discovery rate) at 0.01. Venn diagrams showing the overlap of differentially expressed genes among three data sets including ITD/ITD; *p53*^−/−^ (**d**: downregulated; **e**: upregulated). Pink = ITD/ITD vs WT; Orange = *p53*^−/−^ vs WT; Yellow = ITD/ITD; *p53*^−/−^ vs WT. Venn diagrams show the overlap of differentially expressed genes in 2 pairwise comparisons (ITD/ITD; *p53*^+/−^ vs ITD/ITD = pink; ITD/ITD; *p53*^−/−^ vs ITD/ITD = yellow) (**f**: downregulated, **g**: upregulated). **h** TaqMan assays confirmed the RNA-Seq data in analyzed animals. ITD/ITD: *n* = 3 mice, ITD/ITD; *p53*^+/−^: *n* = 6, ITD/ITD; *p53*^−/−^: *n* = 4, *p53*^+/−^: *n* = 4 (for *Lin28a* and *Sowaha*) or 3 (for *FLT3* and *Htra3*), *p53*^−/−^: *n* = 2. **i** Overexpression of *Htra3* inhibited apoptosis induced by TGF-ß. 32D cells were transduced by a retroviral vector [45] expressing murine *Htra3*, and two clones with almost 100% expression of *Htra3* were selected by limited dilution. Transduced 32D cells were cultured in the presence of TGF-ß without IL-3. Results presented are the mean ± SD of at least 4 independent experiments. **p < 0.01. **j** Representative example of macroscopic spleen colonies (CFU-S) from wild-type irradiated recipients injected with Lin− cells from ITD/ITD donors transduced with *Htra3* or control cells from ITD/ITD mice (left panel). Right panel shows CFU-S from recipients injected with Lin− cells from WT donor. Spleens were fixed in Carnoy’s solution. Note that there were around 20% transplanted cells were with *Htra3* **k**. **l** Represent cytospins showing different morphology of 32D WT and 32D overexpressing *Lin28a* cells (32D-*Lin28a*) on day 2 after G-CSF treatment. About 75% 32D-*Lin28a* cells went to differentiation (myelocytes to segmented neutrophils [45, 46]), while only 11% 32D WT cells differentiated. Similar differences were also observed on day 4, day 6, and day 8 after G-CSF treatment (data not shown). **m** Cytospin from mouse #1884 with erythroid leukemia showing strong infiltration of erythroblasts in spleen (left panel). Cytospin from mouse #1993 with AML showing >50% myeloblasts in BM spleen. Most myeoloblasts showed morphological features of immature monocytes similar as leukemic cells from double-transgenic mice (Fig. 2a). Mice #1884 and #1994 were transplanted with ITD/ITD lin− cells overexpressing *Htra3* and overexpressing *Htra3/Lin28a* knockdown, respectively.

To further understand how p53 haploinsufficiency or loss accelerates the leukemogenesis of FLT3-ITD, we prepared RNA samples from BM cells from WT, *p53*^+/−^, ITD/ITD; *p53*^+/−^, ITD/ITD; *p53*^−/−^, and *p53*^−/−^ mice for comparative RNA-sequencing analysis. We performed hierarchical clustering analysis of 2176 differentially expressed genes (DEGs) after multi group comparison at a corrected *p* value threshold of < 0.01 (Fig. 4a). Furthermore, we expanded the statistical approach to explore meaningful pairwise comparisons by use of DESeq2 [25]. Interestingly, consistently more genes were downregulated than upregulated in all samples compared with the WT (Fig. 4b–e; Table 1; e.g., 1756 vs. 981 in the ITD/ITD group). Notably, compared with the WT, more genes were differentially expressed in mice with double mutations than the sum of single mutations (Fig. 4b–e; Table 1; e.g., 4430 genes in ITD/ITD; *p53*^+/−^ vs WT). These findings suggest a specific interaction between ITD/ITD and *p53*KO.

**Table 1:** Pairwise comparisons revealing the numbers of differentially expressed genes.

Pairwise comparison	Number of differentially expressed genes	Up	Down
ITD/ITD vs WT	2737	981	1756
*p53*^+/−^ vs WT	529	78	451
ITD/ITD; *p53*^+/−^ vs WT	4430	1925	2505
ITD/ITD; *p53*^−/−^ vs WT	5675	2353	3322
*P53*^−/−^ vs WT	2523	762	1761
ITD/ITD; *p53*^+/−^ vs ITD/ITD	1374	780	594
ITD/ITD; *p53*^−/−^ vs ITD/ITD	279	76	203

We further identified the intersection of DEGs explored in two pairwise comparisons (ITD/ITD; *p53*^+/−^ vs ITD/ITD and ITD/ITD; *p53*^−/−^ vs ITD/ITD) and found that 11 and 82 genes were consistently upregulated and downregulated, respectively (Fig. 4f–g; Supplementary Fig. 11). Gene Ontology (GO) analyses revealed several pathways affected by the cooperation of ITD/ITD and *p53*KO that were directly related to hematopoiesis, including myeloid differentiation and development (Supplementary Table 3). To support our data, we also analyzed gene expression in AML patients with *FLT3*-ITD vs AML without *FLT3*-ITD in the publicly available TCGA database [26]. Comparing this dataset with our dataset (ITD/ITD; *p53*^+/−^ vs ITD/ITD and ITD/ITD; *p53*^−/−^ vs ITD/ITD), we found eight genes (*CARHSP1*, *OPTN*, *LIN28A*, *FLT3*, *TMCC2*, *CPEB4*, *FHDC1*, and *HMBS*) in both datasets. We performed quantitative PCR assays for seven selected genes (e.g., *Htra3*, *FLT3*, *Lin28a* and *Sowaha*, which belong to the genes with the most changed expression) from these 93 genes (Fig. 4h; Supplementary Fig. 11), and the assays were correlated very well with the RNA-seq data. TaqMan assay demonstrated that *Htra3* was up to 149-fold upregulated by the co-operation of ITD/ITD and *p53*KO, while *Htra3* was downregulated by any single mutation. The role of serine protease *Htra3* in the leukemogenesis is unknown [27]. Consistent with a report demonstrating *Htra3* as an inhibitor of TGF-ß signaling (a tumor suppressor in human hematologic malignancies) [28], we also found that overexpression of *Htra3* in 32D murine myeloid cells significantly inhibited TGF-ß-induced apoptosis (Fig. 4i). In CFU-S assays, Lin− cells from ITD/ITD donors transduced with *Htra3* gave rise to larger and more numerous CFU-S compared with non-transduced Lin− cells obtained from ITD/ITD donors (Fig. 4j, k, mean colonies/mouse 12 vs. 2, *p* < 0.05, *n* = 3 and 5, respectively), whereas no increase in the CFU-S number was observed for Lin− cells from WT donors that were transduced with *Htra3* compared with control cells (*n* = 3 for each). This result indicates strong cooperation between *FLT3*-ITD and *Htra3* in support of multipotent progenitor proliferation, although we did not find any differences in the proliferation capacities between ITD/ITD; p53^+/−^ and ITD/ITD lin^−^ cells in vitro (data not shown). *Lin28a* was up to 14-fold downregulated by cooperation of ITD/ITD and *p53*KO. Moreover, the immortalized 32D myeloid cells showed 231-fold reduced expression of *Lin28a*. Overexpression of *Lin28a* in 32D cells strongly promoted G-CSF induced differentiation (Fig. 4l). In murine models, miR-125b overexpression leads to downregulation of *Lin28a* and the development of myeloid leukemia [29]. Although cultured ITD/ITD cells overexpressing *Htra3* did not result in an increase in lin^−^ cells, the knockdown of *Lin28a* in ITD/ITD cells resulted in a 1.5-fold increase in lin− cells after culture in vitro, indicating the inhibition of differentiation by *Lin28a* knockdown. In the presence of overexpressed *Htra3*, lin^−^ cells with *Lin28a* knockdown increased up to 5-fold after culture for over 7 days compared with control cells, indicating a synergistic effect between *Lin28a* and *Htra3* in the differentiation inhibition of ITD/ITD cells. Moreover, we observed AML (erythroid leukemia) development in the group transplanted with ITD/ITD lin^−^ cells overexpressing *Htra3* 5 months after transplantation (one of five mice, cut off: 1st of August 2021), and the combined knockdown of *Lin28a* and overexpression of *Htra3* in ITD/ITD cells induced AML (two of three mice) with a much shorter latency of one month and a similar morphology as leukemic cells obtained from ITD/ITD; *p53* KO double-transgenic mice. The third mouse did not show sign of leukemia about two months after transplantation. By contrast, no sign of leukemia was observed in six mice transplanted with ITD/ITD lin^−^ cells with *Lin28a* knockdown 4 months after transplantation, indicating a cooperative effect between *Htra3, Lin28a*, and ITD/ITD in AML induction. We isolated BM cells from both young non-diseased ITD/ITD; *p53*^*+/−*^ mice and old leukemic ITD/ITD; *p53*^*+/−*^ mice and transplanted these cells into WT recipients. No significant difference was observed in survival between these 2 groups (149 vs 143 days, *n* = 3 for each), suggesting that a contribution from the BM microenvironment to leukemia development in our model is unlikely.

Importantly, consistent with the lack of enhancement of aberrant self-renewal by the co-occurrence of *FLT3*-ITD and *p53* KO, we observed opposite expression of *Arhgap5* and *Lin28a* that are known for the requirement of HSC self-renewal [30, 31]. Interestingly, overexpression of *Htra3* in HSCs/HPCs carrying *FLT3*-ITD did not increase replating activity in vitro, suggesting no increased self-renewal activity by overexpression of *Htra3*. Moreover, we found over 30-fold upregulation of FLT3 by the co-occurrence of *FLT3*-ITD and *p53* KO. The loss of self-renewal capacity by upregulation of *FLT3* expression in the bone marrow LSK stem cell compartment has been reported previously [32]. Taken together, our data indicate that *FLT3*-ITD and *p53* KO synergistically enhance the expression of *Htra3* and reduce the expression of *Lin28a*, leading to leukemia development by increased proliferation, reduced apoptosis, and enhanced block of differentiation, but without increasing the self-renewal activity.

### Potent activity of carfilzomib combined with R428 against preclinical models of AML

**Fig. 5:**
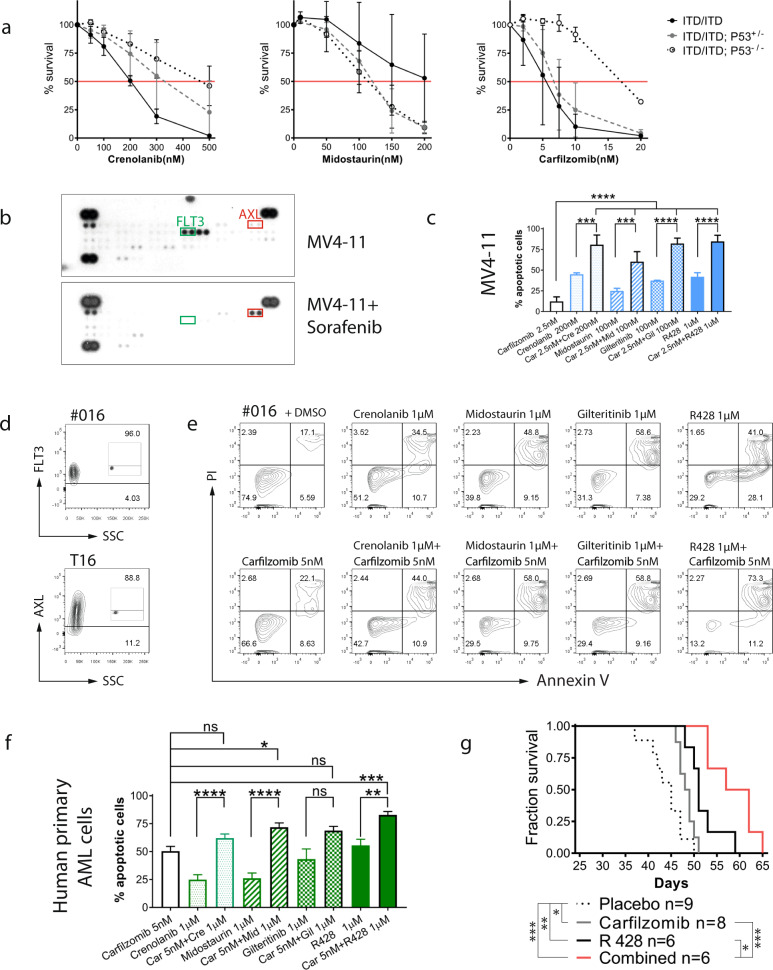
Development of efficient therapies for ITD/ITD; *p53* KO leukemia. **a** Growth inhibition of ITD/ITD, ITD/ITD; *p53*^+/−^ and ITD/ITD; *p53*^−/−^ leukemic cells treated with crenolanib, midostaurin, and carfilzomib. Of note, ITD/ITD; *p53*^−/−^ leukemic cells were highly sensitive to carfilzomib (IC50: 17.1 nM), although ITD/ITD; *p53*^−/−^ leukemic cells appeared to have decreased sensitivity to carfilzomib compared with ITD/ITD and ITD/ITD; *p53*^+/−^ cells (IC50: 5.2 nM and 6.8 nM, respectively). Apoptosis was analyzed by Annexin V and propidium iodide (PI) staining. Leukemic cells were treated with different concentrations of crenolanib, midostaurin, and carfilzomib for 48 h before flow cytometric analyses. Survival was defined as Annexin V/PI double-negative staining. Apoptosis was defined as Annexin V-positive or PI-positive staining. Each plot and bar represents the mean viability rate ± SD under treatment with the indicated inhibitors. T-ALL cell lines, but no AML cell lines were generated from individual mice. ITD/ITD, *n* = 6; ITD/ITD; *p53*^+/−^, *n* = 6; and ITD/ITD; *p53*^−/−^, *n* = 3. **b** MV4-11 and MV4-11 treated with Sorafenib were simultaneously subject to an antibody array. Treatment with Sorafentib significantly dephosphorylated FLT3 and induced strong AXL activation. Specificity for the phosphorylation of AXL was confirmed by western blot (Supplementary Fig. 13). **c** Effects of combined therapies in the MV4-11 AML cell line with *FLT3*-ITD, showing the 20-fold and 4-fold increased expression of *MDM2* and *MDM4*, respectively. The results presented are the mean ± SD of at least three independent experiments. Combination index: carfilzomib/crenolanib = 0.36, carfilzomib/midostaurin = 0.25, carfilzomib/gilteritinib = 0.39, carfilzomib/R428 = 0.36. **d** Flow cytometric analyses showing the expression of *FLT3* and *AXL* in patient #016. The negative controls were shown as inserts. AXL expression was also found in five of six myeloid leukemic cell lines (data not shown). **e** A representative flow cytometric analysis showing the strong induction of apoptosis, induced by the combinations of carfilzomib/midostaurin and carfilzomib/R428 in primary AML cells from patient #016 with *FLT3*-ITD and up to four-fold increased expression levels of *MDM2* and *MDM4*. **f** Effects of combined therapies in primary patient samples. The results are presented as the mean ± SD of independent experiments. The combination of carfilzomib/R428 induced more apoptotic cells than any single treatment in all patient samples, whereas for other combined therapies an inferior effect was observed in some patient samples when compared with single treatments. For example, carfilzomib/crenolanib induced fewer apoptotic cells than carfilzomib in five patient samples. Up to 25-fold and 41-fold increased expression levels for *MDM2* and *MDM4*, respectively, were observed in samples from *FLT3* + patients by TagMan assays. Only one patient harbored a *TP53* mutation, for which no additive/synergistic cytotoxicity was observed in the combined therapies. We took both *FLT3*-ITD and *FLT3* WT AML samples for analyses because proteasome inhibitors can downregulate the protein expression of both *FLT3*-ITD and *FLT3* WT [47], and *AXL* may play an important role in the pathogenesis of *FLT3*-ITD and *FLT3* WT AML [34]. Carfilzomib: *n* = 22 patients; crenolanib: *n* = 22; midostaurin: *n* = 22; gilteritinib: *n* = 11; R428: *n* = 14; carfilzomib/crenolanib: *n* = 22; carfilzomib/midostaurin: *n* = 22; carfilzomib/gilteritinib: *n* = 11; carfilzomib/R428: *n* = 14. *p < 0.05, **p < 0.01, ***p < 0.001, ****p < 0.0001. **g** Survival curve of NSG mice transplanted with MV4-11 cells. Therapies were started on day 3 and stopped on day 23 (with the transplantation date representing day 0). *p < 0.05, **p < 0.01, ***p < 0.001.

Interestingly, we found that p53 haploinsufficiency or loss reduced the sensitivity of murine *FLT3*-ITD leukemia to crenolanib in vitro but did not reduce their sensitivity to midostaurin (Fig. 5a). Notably, the combination between *FLT3* inhibitors and the *MDM2* antagonist idasanutlin or the *BCL2* inhibitor venetoclax did not enhance the induction of apoptosis in leukemic cells from ITD/ITD; *p53*^+/−^ mice (data not shown). While *p53* missense mutants cooperate with *Nrf2* (NFE2L2) to activate proteasome gene transcription [33], resulting in resistance to the proteasome inhibitor carfilzomib, our comparative RNA-sequencing analysis did not reveal the activation of proteasome gene transcription by *p53* KO. Consequently, exposure to carfilzomib showed a strong cytotoxic effect against ITD/ITD; *p53*^+/−^ and ITD/ITD; *p53*^−/−^ leukemic cells (Fig. 5a). Carfilzomib enhanced the cytotoxicity of crenolanib, midostaurin (both *FLT3* inhibitors) or R428 (targeting *AXL*, the upstream kinase of *FLT3* [34]) in murine leukemic cell lines and primary murine leukemic cells from ITD/ITD; *p53*^+/−^ and ITD/ITD; *p53*^−/−^ mice (Supplementary Fig. 12 and data not shown).

We and others have obtained evidence to support a potential role of *AXL* in the pathogenesis of AML [35, 36]. In this study, we found that myeloid progenitor 32D cells were easily immortalized by AXL activation (Supplementary Fig. 13 and data not shown), suggesting a potential transforming activity for *AXL* in hematopoietic cells. Moreover, the inhibition of FLT3 led to the strong activation of AXL (Fig. 5b), which supported earlier reports that suggested drug resistance induced by AXL activation [37]. MV4-11 cells with wt-p53 were sensitive to all combined treatments, including carfilzomib/R428, in vitro (Fig. 5c). Low combination index (<0.4) for all combinations indicate a strong synergistic cytotoxicity in MV4-11 cells. We then analyzed expression and activation of AXL in primary samples from AML patients. The expression of *AXL* and the strong phosphorylation of AXL were found in 74% (37/50) of primary cells by flow cytometric analysis and in ~31% primary samples (28/90) by phospho-kinase antibody array, respectively (Fig. 5d, and data not shown). Almost all leukemic cells with expression of *AXL* were also positive for *FLT3* (Fig. 5d). We then treated primary leukemic cells from AML patients with or without FLT3-ITD with combinations of carfilzomib and FLT3/AXL inhibition in vitro (Fig. 5e). Totally, all combination therapies induced more apoptotic cells than single therapies (Fig. 5f). However, a significant difference was only observed for carfilzomib /R428 and carfilzomib/midostaurin combinations. Moreover, we also treated samples from 2 AML patients with FLT3-ITD, who relapsed after treatment with FLT3 inhibitors. These leukemic cells were also highly sensitive to carfilzomib and combination with FLT3i/AXLi with a combination index of 0.98 for carfilzomib /R428 combination in one sample. Because monotherapy with carfilzomib or R428 showed only moderate effects in patients with AML [38], we tested the therapeutic effects of the carfilzomib/R428 combination in a xenotransplantation model in vivo. While all of the examined therapies significantly prolonged the survival of mice transplanted with MV4-11 cells, the combined therapy of carfilzomib and R428 was more efficient than any monotherapies with carfilzomib or R428 (Fig. 5g). The mean plasma concentrations of carfilzomib and R428 were among the therapeutic windows reported, which were for example 227 nM at 5 min and 5.6 µM at 6 h after treatment, respectively (Supplementary Table 4). Collectively, carfilzomib and R428 combination led to a strong additive/synergistic cytotoxicity in leukemic cells. Our data suggest that the combination of carfilzomib and R428 or *FLT3* inhibitors (e.g., midostaurin) might represent promising treatment options, particularly in *FLT3*-ITD positive AML.

## Discussion

Our study presents a powerful synergy between FLT3-ITD and p53 haploinsufficiency or loss in the induction of AML and emphasizes more careful analysis of p53 deregulation in AML. Our murine model is highly consistent with human disease. With an incidence of ~50%, absent or reduced p53 protein levels are common in AML. Approximately 5% of all AML patients feature a p53 deletion, whereas the frequency of p53 loss has been reported in 29% of patients with a complex aberrant karyotype [39]. Although the biallelic loss of *TP53* is a rare event in AML, the *TP53* tumor suppressor gene is frequently inactivated in tumors through a two-hit mechanism, in which one allele carries a missense mutation and the other allele is lost by the deletion of human chromosome 17p, which may give rise to the complete loss of wt-p53 function. Although the co-occurrence of a p53 deletion and FLT3-ITD is not common in AML, AML associated with *FLT3*-ITD mutations is highly associated with wt-p53 dysfunctions via various mechanisms [11]. For example, patients with *FLT3*-ITD and *NPM1* mutations showed a 98% reduction in functional nuclear p53 protein compared with healthy controls [10]. Collectively, the reduced dosage of p53 is common in AML with *FLT3*-ITD. Our model provides proof-of-principle that demonstrates the important role of reduced p53 dosage in the pathogenesis of *FLT3* + AML. In ongoing studies, we plan to explore whether *MDM2* or *MDM4* overexpression in the presence of *FLT3*-ITD can induce AML. Because p53 is regulated/modified by many non-genetic factors, analyzing the cooperation between a single non-genetic factor and *FLT3*-ITD during the induction of AML might be difficult. Notably, overexpressed p53 has also been found in the normal diploid karyotype, particularly in FLT3 mutant patients, and is associated with worse relapse-free survival [40]. Whether overexpressed p53 also promotes the development of FLT3 + AML remains to be determined.

**Fig. 6:**
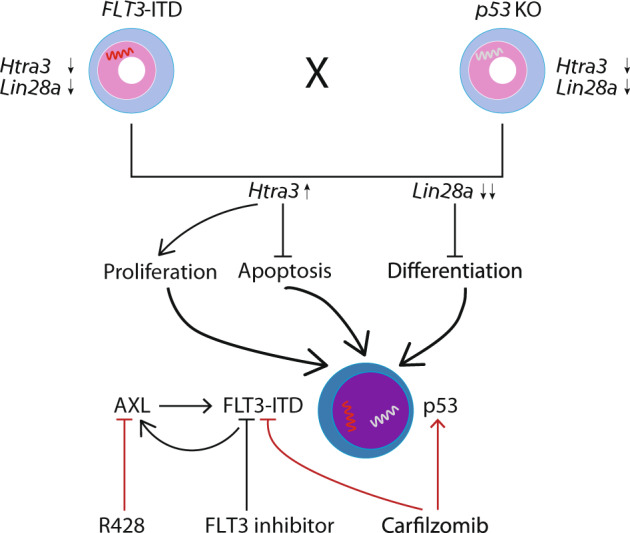
Proposed model for the cooperation of FLT3-ITD and p53 haploinsufficiency or loss, and combined therapy. The cooperation of FLT3-ITD and p53 haploinsufficiency or loss synergistically induced the downregulation of *Lin28a* and the upregulation of *Htra3*, which led to increased proliferation, the inhibition of differentiation and apoptosis, and leukemia development. *Htra3* is likely a central mediator, through inhibition of apoptosis and enhancement of proliferation [48]. Moreover, *Htra3* also enhanced the inhibition of differentiation by *Lin28a*. Of note, *Htra3* expression in normal HSCs/HPCs is not increased and similar as other mature blood cells (http://servers.binf.ku.dk/bloodspot/?gene). Proteasome inhibitors induced the cell death of *FLT3*-ITD-positive AML cells through the autophagy of *FLT3*-ITD and in part the activation of the p53 pathway [47]. Thus, cells with p53 loss can still be sensitive to proteasome inhibitor. AXL can positively regulate the constitutive activation of *FLT3*-ITD [34], whereas the inhibition of FLT3-ITD by FLT3 inhibitors can induce increased expression/activation of AXL (Fig. 5b), which might induce resistance to *FLT3* inhibitors. The overexpression of AXL has been shown to mediate resistance to other kinase inhibitors [49].

Of note, our RNA sequencing analyses indicate a specific signaling interaction between ITD/ITD and *p53* KO (Fig. 4c–j; Table 1). We propose that ITD/ITD and *p53* KO synergistically induce AML via the upregulation of *Htra3* and the downregulation of *Lin28a* (Fig. 6). *Htra3* inhibited apoptosis, specifically promoted the proliferation of multipotent hematopoietic progenitors bearing *FLT3-IT*D in vivo, and enhanced the inhibition of differentiation by *Lin28a*. The upregulation of *Htra3* and the downregulation of *Lin28a*, combined with the presence of ITD/ITD, induced AML with similar morphology as leukemic cells from double-transgenic mice. The extremely short latency of about one month suggest that other molecular event may not be required for AML induction by these three events, although the analyzed cases are limited. Because the role of serine proteases *Htra* in the leukemogenesis is largely unknown [27], we are currently investigating, in greater detail, the roles of *Htra3* and its interaction with *Lin28a* in leukemogenesis in ongoing studies. It will be important to determine the expression of *Htra3* and its impact on prognosis in a large cohort of patients with FLT3mut AML and dysfunction of wtp53. Moreover, the role of other upregulated or downregulated genes (e.g., Sowaha) in hematopoietic cell differentiation and leukemia development remains to be determined. The cooperation of FLT3-ITD and p53 haploinsufficiency or loss demonstrates some unique features. While p53 mutants may enhance the self-renewal activity and induce an abnormal karyotype [41], p53 haploinsufficiency or loss in the presence of FLT3-ITD enhanced proliferation and the blockade of cell differentiation, and reduced apoptosis rather than enabling the aberrant self-renewal activity and chromosomal instability. We demonstrate a clear difference between mutated p53 and reduced p53 dosage in terms of their contributions to *FLT3* + AML development. While p53 haploinsufficiency or loss strongly promoted the leukemogenesis of *FLT3*-ITD (shortened survival and high incidence of AML, Fig. 1), Nabinger et al reported that *p53*^*R248W*^ mutation did not cooperate with *FLT3*-ITD to induce AML and to reduce the survival of mice [6]. Further studies remain necessary to elucidate the mechanisms through which *p53* haploinsufficiency or loss exerts different functions than *p53* mutations in the presence of FLT3-ITD.

Finally, our results may be relevant to the treatment of acute leukemia. Our data suggest that a clinical trial examining the combination of carfilzomib and R428 in AML patients could provide insightful new data. Because dysfunction of the p53 pathway by other reasons, such as the overexpression of *MDM2/MDM4*, is also often found in other human cancers [1], therapies containing carfilzomib might be an effective strategy for other human cancers with reduced p53 dosage or loss.

## Supplementary information

Suppl. data
