# Supplementary material for: A unique role of p53 haploinsufficiency or loss in the development of acute myeloid leukemia with FLT3-ITD mutation
Source: Leukemia. 2021 Nov 3;36(3):675–86. doi: 10.1038/s41375-021-01452-6 (PMC8885416; doi:10.1038/s41375-021-01452-6)
Supplement: Supplementary file 1 — Suppl. data [file 41375_2021_1452_MOESM1_ESM.docx]

**Supplementary data (Yang M et al.)**

**SUPPLEMENTARY METHODS**

***FLT3*-ITD; *p53* knockout mice**

We chose homozygous ITD/ITD mice to examine the cooperating function of FLT3-ITD and p53 dysfunction for several reasons, for example: 1) we reported a differential impact of the allelic ratio and insertion site in *FLT3*-ITD-positive AML with respect to allogeneic transplantation. Multivariable analyses revealed a high allelic ratio as a predictive factor for the beneficial effect of allogeneic HSCT [1]. 2) Most human AML cell lines with ITD have ITD/ITD, e.g., MV4-11 [2]. 3) Loss of the WT allele strongly enhanced the oncogenic potential of *FLT3*-ITD as reported previously (Supplemental Fig. 2 and 3) [3].

For comparison of the survival time and disease spectrum, we took 76 mice in the ITD/ITD; *p53*^+/-^ group. In this group, 56 mice had at least one littermate/sibling in the ITD/ITD group. However, for the other 20 mice, there was no ITD/ITD littermate/sibling or no data on long-term survival available. Because all ITD/ITD; *p53*^+/-^ mice had a common ancestor and no significant difference was found between these two ITD/ITD; *p53*^+/-^ subgroups (with or without ITD/ITD littermates/siblings) in terms of the mean survival and AML incidence, therefore we combined these two subgroups together. Wildtype C57Bl/6J mice were used as control mice.

# Analysis of hematopoietic stem/progenitor compartments

# To analyze the frequencies of different hematopoietic stem/progenitor populations, single-cell suspensions of BM and spleen (in some cases) were stained with a mouse hematopoietic lineage antibody cocktail (mixture of Anti-mouse CD3 FITC, Anti-mouse CD45R (B220) FITC, Anti-mouse CD11b FITC, Anti-mouse TER-119 FITC, Anti-mouse Ly-G6 (Gr-1) FITC) (eBioscience #22-7778-72), Anti-mouse CD127 FITC (eBioscience #11-1271-82), Anti-mouse c-Kit PE (eBioscience #12-1172-82), Anti-mouse Ly-6A/E (Sca-1) PE-cy7 (eBioscience #25-5981-82), Anti-mouse CD150 APC (eBioscience #17-1502-80), Anti-mouse CD48 APC-eFluor780 (eBioscience #47-0481-82), Anti-mouse CD127 APC-eFluor780 (eBioscience #47-1271-82), Anti-mouse CD34 AF-647 (Biolegend # SA376A4), Anti-mouse CD16/32 (Fcgr3/ Fcgr2) APC-cy7 (Biolegend #101328) for 30min in dark. 7-AAD (Biolegend #420403) was added to exclude dead cells 5 mins before acquisition.

**Genomic DNA Isolation and Genotyping**

Genomic DNA was isolated from either ear biopsy or single-cell suspensions by using the DNA blood mini kit. For the genotyping analysis of FLT3-ITD and wild-type FLT3, the forward primer (5’-AGGTACGAGAGTCAGCTGCAGATG-3’) and reverse primer (5’-TGTAAAGATGGAGTAAGTGCGGGT-3’) were used to amplify the FLT3 gene by PCR. For the genotyping analysis of wild-type p53 and p53 knockout, the forward primer against exon 6 (5'-ACAGCGTGGTGGTACCTTAT-3'), reverse primer against exon 7 (W3: 5'- TATACTCAGAGCCGGCCT-3') and forward primer against neo (5'- CTATCAGGACATAGCGTTGG-3') were used. The PCR products were submitted to agarose gel electrophoresis.

**Immunophenotypic analysis**

In total, 1×106 cells of each organ were washed with PBS and suspended in 100 μl of FACS buffer and then were stained with antibodies for 30 min away from light at 4°C. Leukemic cells were stained at least with immature myeloid markers (c-Kit and CD34), mature myeloid markers (Gr-1, CD11b), an erythrocyte marker (TER119), and lymphocytic markers (CD3, CD4, CD8 and CD19). After incubation, the cells were washed with PBS and resuspended in 400 μl of FACS buffer for acquisition by a FACS machine. Propidium iodide (PI) was supplemented immediately before acquisition to exclude dead cells when necessary. Measurement of cell surface markers was performed using the BD FACS Canto or BD FACS Canto II system.

**Multicolor fluorescence in situ hybridization (mFISH)**

mFISH analysis was performed in selected mice as described previously [4]. At least five metaphases from each sample (>10 in most cases) were analyzed. In some mice, samples from two organs (bone marrow, spleen, or thymus) were used.

# Small-molecule inhibitor treatment

# Leukemic cells were cultured in IBM supplemented with 10% FBS at the concentration of 10^5^ cells/ml. Cell viability was analyzed using the Annexin-V assay (BD Pharmingen, Heidelberg, Germany). The cells were generally treated with the indicated inhibitors for 48 h, were stained with Annexin-V FITC/APC (Biolegend, San Diego, CA) for 15 min and then were washed with 500μl of Annexin V binding buffer. PI was added immediately before FACS acquisition. At least 10,000 cells were measured for each sample. Annexin V+/PI- cells were considered as early apoptotic cells; Annexin V+/PI+ cells were considered as late apoptotic cells; Annexin V-/PI+ cells were considered as dead cells. Survival cells were considered to be Annexin V-/PI-. Cell viability was normalized to cells treated with DMSO. Primary murine AML cells were suspended in Iscove Basal Medium containing 20% FBS at the concentration of 2.5×10^5^/ml to 5×10^5^/ml and were treated with inhibitors immediately after single-cell suspensions were made. Apoptotic assays were performed after 24-h incubation. Combination index was calculated by using compuSyn software.

**Cell growth in methylcellulose and serial replating assays**

To analyze clonal growth, 10^4^ BMMCs from selected mice were plated in GG M3434 methylcellulose media with cytokines (SCF, EPO, TPO, IL3, and GM-CSF; StemCell Technologies, Vancouver, Canada). The cells were plated as duplicates or quadruplicates. Colonies were counted after 10-12 days of initial culture, and all cells were washed with IMDM and collected, and then were subsequently plated at 10^4^ cells per dish in the same media. Colony counting and replating were performed weekly for at least three times or until no colonies grew in the media. Proliferation assay was performed by using Click-iT EdU (5-ethynyl-2’-deoxyuridine) kit (Thermo Fisher, Waltham, MA, USA), according to the manufacturer’s protocol.

**Limiting dilution transplantation of leukemic cells and CFU-assay**

We performed the limiting dilution assay to check the frequency of leukemic stem cells (LSCs) *in vivo*. For limiting dilution transplantation of leukemic cells, 10^6^, 10^5^, 10^4^, 10^3^, 10^2^, 10, 2 and 1 cultured leukemic cells from diseased mice were transplanted into secondary recipients (generally 2-3 mice/cell dose). In this study, the cell dose of each group was compared, with which 100% leukemia development in the recipient mice was induced [5]. Irradiated animals were transplanted with gene-modified cells. Around twelve days later the mice were euthanized and well-separated, discrete splenic colonies were carefully dissected and a single cell suspension was prepared.

**Retroviral vectors and transduction**

Murine *Htra3* cDNA was cloned into our retroviral backbone [6]. A retroviral vector expressing murine *Lin-28a* was purchased from Addgene (Watertown, MA). Retroviral transduction was performed as described previously [6,7].

**Antibody arrays and patient samples**

Human RTK antibody arrays were performed according to the manufacturer’s protocol but with 2 mg protein lysate per array [8]. We studied tumor specimens from AML patients who had been diagnosed in the Hannover Medical School. Blood and/or bone marrow (BM) samples from AML patients were collected at diagnosis after informed consent. Mononuclear cells from all samples studied were immediately isolated by centrifugation over Ficoll gradient and freshly used or stored at –180^0^C until further use. Studies with human samples were approved by the ethical committee of Hannover Medical School.

# Xenograft studies

For the xenograft studies, NSG (Mus musculus_NOD.Cg-Prkdc^scid^ II2rg^tm1wjl^/SzJ, The Jackson Laboratory) mice were irradiated with 2.5Gy and transplanted with MV4-11 cells. We chose 10^6^ MV4-11 cells/mouse for transplantation, since animals transplanted with ≥10^6^ cells survived around 36 days after transplantation in our preliminary study. R428 (bemcentinib=BGB324, MedChemExpress, Monmouth Junction, NJ) was dosed BID, 25 mg/kg, 5 days on, 2 days off per week. R428 was reconstituted and prepared as previously published [9]. Carfilzomib (Sellleckechem, Houston, TX) was given 5mg/Kg QD on 2 days per week together with R428.

**SUPPLEMENTARY DISCUSSION**

Why does the loss of *p53* (or heterozygosity) have a different function in the presence of *FLT3*-ITD compared with a *p53* mutation? p53 haploinsufficiency or loss may have a differential impact on cell differentiation and proliferation than mutated p53. Mutant p53 did not affect myeloid and lymphoid differentiation following HSC transplantation [10]. The *p53^R248W^* mutation in the presence of *FLT3*-ITD only induced an increase in GMP but not CMP [11]. In contrast, we observed a strong increase in CMP but not GMP in our model (Fig. 3a). Although both CMP and GMP can serve as cells of origin for leukemia in AML, it has been shown that more oncogenes can transform CMPs [12], which suggests that the less differentiated CMPs are likely easier to be transformed and initiate AML than the more mature GMPs. We observed the strong upregulation of *Htra3* in *FLT3*-ITD and *p53* KO, and *Htra3* in the presence of *FLT3*-ITD strongly supported the proliferation of multipotent progenitor cells *in vivo* (Fig. 4j). Moreover, *p53* KO in the presence of *FLT3*-ITD resulted in the downregulation of *Lin28a*, leading to the inhibition of differentiation. Another difference between mutant p53 and p53 haploinsufficiency or loss is the ability to interact with EZH2. Mutant p53 interacts with EZH2 and enhances its association with chromatin, increasing the levels of H3K27me3 in the genes that regulate HSC/HPCs self-renewal and differentiation [10], whereas EZH2 target gene signature was not significantly changed by loss of *p53* in HPCs and *p53* KO may affect neither EZH2 activity nor H3K27me3 in HPCS [10]. Consistently, we did not observed significant change of EZH2 target gene signature by the combination of ITD/ITD and *p53* KO (data not shown). Whether this difference and differently expressed p53 target genes (Fig. S11) might contribute to the different functions (and phenotypes) observed for mutated p53 and p53 haploinsufficiency or loss in the presence of FLT3-ITD remains unclear. Notably, the reduced activity and loss of EZH2 is a common characteristic of AML, and the loss of EZH2 has been associated with poor prognosis and chemoresistance in AML [13].

Interestingly, HTRA3 expression was most profoundly changed in ITD/ITD; *p53^+/-^*, where *Lin28a* expression was most profoundly reduced in ITD/ITD; *p53 ^-/-^* mice (Fig. 4h). The reason for this is unclear. Interestingly, a few mice in the ITD/ITD; *p53^+/-^* group and #1884 mouse transplanted with ITD/ITD lin- overexpressing *Htra3* developed erythroid leukemia, while we did not observe erythroid leukemia in the ITD/ITD; *p53 ^-/-^* group. Whether high expression of *Htra3* promotes development of erythroid leukemia remains to be determined.

In this study, for the first time, we identified the activation of AXL in a large proportion of AML patients using an antibody array. We did not observe any changes in *Axl* expression in murine leukemic cells with ITD/ITD and *p53* KO by RNA-seq analysis, which suggested that *Axl* may not be directly involved in AML development in our model. However, *Axl* appears to be important for the survival of these leukemic cells, as these cells were highly sensitive to R428 treatment. Because AXL signaling has been shown to regulate the function of cancer stem cells, including CML [14], breast cancer [15], the investigation of whether *AXL* is required for the maintenance of LSC in *FLT3*+ AML remains necessary.

Leukemic cells induced by *FLT3*-ITD and *p53* knockout did not demonstrate increase of the self-renewal potential (Fig. 2f; supplemental Table 2). This may explain, at least in part, why the latency of AML in our model is longer than that induced by cooperation of *NPM1c* or *Dnmt3a* with *FLT3*-ITD associated with an aberrant self-renewal potential [16-18], although an additional event in our model cannot be ruled out. In one study, DNMT3A loss in cooperation with ITD/ITD mainly induced ALL and did not shorten the survival of animals compared with DNMT3A loss alone [17]. AML induced by NPM1c cooperated with FLT3-ITD was in part due to wt-p53 dysfunction by MDM2-mediated degradation of p53 [19]. Therefore, our data indicated that acute leukemia may also develop in the absence of enhanced self-renewal of HSC. p53 loss cooperated with KRAS^G12D^ to induce AML by enabling aberrant self-renewal of HSC [20], while there was no evidence for enhanced self-renewal in our mice with both *p53* KO and *FLT3*-ITD. These indicate that p53 haploinsufficiency or loss displays context-dependent roles in the pathogenesis of AML. One limitation of our study is the use of constitutive deletion of *p53* in transgenic mice. The incidence of AML in our double-mutant mice would be higher if conditional knockout of *p53* (e.g., in myeloid lineage) was used, because many mice were analyzed due to lymphoma (enlarged thymus), while myeloblasts in the bone marrow increased but had no chance to reach 20% or more. Moreover, although we cannot rule out a contribution from the BM microenvironment to leukemia development in our model, our data (e.g. Fig. 4) indicate that the intrinsic mechanisms of ITD/ITD; p53^+/−^ cells rather than the BM microenvironment drives the development of acute leukemia and accelerates disease progression.

Because over half of ITD/ITD; p53^+/−^ mice developed ALL (alone or as a biclonal disease with the coexistence of AML and ALL/lymphoma), our data also indicated a strong cooperative effect between FLT3-ITD and p53 haploinsufficiency in the induction of ALL. In contrast, the co-occurrence of p53 mutations and FLT3-ITD did not induce ALL in a mouse model [11]. Because *FLT3* mutations have been reported in patients with T-ALL and B-lymphoblastic leukemia, with an incidence of up to 25% in some subtypes [21], our data (Supplemental Fig. 12) also suggested that the combination of carfilzomib and R428 or *FLT3* inhibitors (e.g., midostaurin) might be an efficient treatment option for ALL patients with *FLT3* mutations or high levels of FLT3 expression.

**SUPPLEMENTARY TABLES AND FIGURES**

**a**

**b**


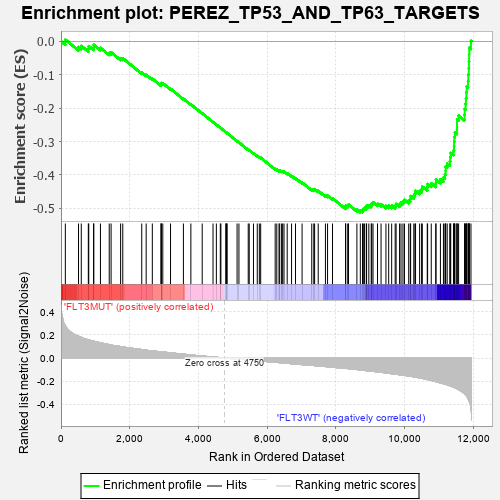


**Fig. S1 Overexpressed *MDM2* and *MDM4* in *FLT3*-mutated AML patients.** **a** heterogeneous expression of *MDM2* and *MDM4* mRNA in *FLT3*-mutated AML. **b** Gene enrichment analysis, correlating the *FLT3*-mut AML transcriptome with downregulated *TP53* targets (NES: −1.8807226, NOM p-val: 0.001828154, FDR q-val: 0.1506825). Data for AML patients was obtained from the TCGA database [22] and analyzed by cBioPortal ([http://cbioportal.org](http://cbioportal.org/)) [23,24]. Overexpressed cases were defined as those with mRNA expression levels above the mean of the AML cohort.

**Fig. S2 *FLT3*-ITD mice primarily developed fatal MPN (CMML-like disease).** The loss of the WT allele enhanced the oncogenic potential of *FLT3*-ITD. **a** Cytospins of the spleen, demonstrating a normal hematopoietic compartment (approximately 90% lymphocytes) in an ITD/WT mouse (#24) (left panel). Mouse #24 presented normal liver histology (right panel). **b** Cytospins of the spleen, demonstrating strongly increased monocytes and neutrophils in an ITD/ITD mouse (#50). Histology, showing the strong infiltration of myelomonocytes in the liver. Mice #24 and #50 were analyzed 2 months after birth. **c** To investigate the impacts of the loss of the WT *FLT3* allele on disease progression, littermates/siblings of ITD/ITD (*FLT3*-ITD homozygous), and heterozygous *FLT3*-ITD/WT (referred to as ITD/WT) were used. Kaplan-Meier-plots of ITD/ITD and ITD/WT mice (littermates and siblings, mean survival time: 517 vs 781 days, p < 0.01). **d** *FLT3*#524 developed a CMML-like disease. Genotyping by PCR showed that *FLT3*#524 was a homozygote ITD/ITD mouse (left panel). Blood smear showed increased monocytes (right panel). Multicolor fluorescence *in situ* hybridization (mFISH) analysis showing a normal karyotype for the BM cells from mouse #524 (lower panel). **e** *FLT3*#562 developed T-ALL. Histology showing the destroyed structure of the thymus and the infiltration of tumor cells. Thymus cytospin, showing immature lymphoblastic cells. mFISH analysis showing trisomy 1 (41 XX, Ts1[4]/40, XX, inc[2]) in analyzed leukemic cells from the thymus. All analyzed *FLT3*-ITD mice without lymphoma/lymphoblastic leukemia (n = 5) showed a normal karyotype, whereas all analyzed animals with lymphoma/lymphoblastic leukemia (n = 3) showed abnormal karyotypes in tumor samples. This result suggested that *FLT3*-ITD alone is sufficient for the development of a CMML-like disease but requires cooperating events for the development of lymphoma/lymphoblastic leukemia.

**Fig. S3 Progression of myeloid proliferation after serial bone marrow transplantation.**

For transplantation, bone marrow cells from ITD/ITD and ITD/WT mice were isolated and transplanted immediately into irradiated recipients. Mouse #1135, one of three recipients of cells from primary mouse #50 (ITD/ITD, **Fig. S2**), was killed on day 158 due to signs of malignancy. Histopathology analysis showed the increased infiltration of leukemic cells in the liver **a**, increased erythroid cells, and a cluster of megakaryocytes (a classical future of MPD) in the spleen. (**b,c). d** Blood smears showed increased myelomonocytes (normally < 20% of blood leukocytes). **e** Cytological analysis revealed that 90% were erythroid cells, and 10% were myeloid cells, with >20% blasts among myeloid cells. A light increase in myeloid cells and reduced erythroid cells was observed in bone marrow (**c** and **f**). Mice transplanted with ITD/ITD cells showed significantly shorter survival times than mice transplanted with ITD/WT cells (mean survival 172 days vs. 435 days).

**Fig. S4 Development of solid tumors in *p53*^+/−^ mice.** (**a** and **b**) Mouse #1381 (**Fig. 2a**) developed adenocarcinoma in the anus (**a**, ×100) and osteosarcoma (**b**, ×100). **c** The development of adenocarcinoma in mouse #257 (×100). **d** mFISH analysis showing a complex karyotype in tumor cells from mouse #257:

40~72,XXXX,+X,+1,-2,der(4)t(4;10)t(10;11)t(11;?2),der(5)t(5;13),+6,der(6)t(6;9),add(7)(q?),der(7)t(7;19),der(10)t(10;9)t(9;6),der(11)t(11;12),-12,+15,+15,der(15)t(15;9),der(17)t(6;17)x2,der(19)t(19;17)[cp14]

However, mFISH analyses with splenic cells (no tumor infiltration) from two other *p53*^+/−^ mice showed a normal karyotype (data not shown), suggesting that *p53* KO, per se, may not directly induce a complex karyotype but may cooperate with genetic instability to induce solid tumor development.

**Fig. S5 Development of biclonal leukemia (T-ALL in thymus, spleen, and liver, AML in BM) in ITD/ITD; *p53*^−/−^ mice.** Mouse #1352 developed AML in the bone marrow and T-ALL in the thymus.

**Fig. S6** **Representative immunophenotypic analyses of diseased mice**. **a** Expression of Gr1 and CD11b in the BM cells from mice #1436 (ITD/ITD, CMML-like disease), #1381 (*p53*^+/−^), #1376 (ITD/ITD; *p53*^+/−^, AML), and #1348 (ITD/ITD; *p53*^+/−^, AML/T-ALL). **b** No infiltration of lymphoblastic cells in the BM. **c** (left panel): intercellular staining confirmed no infiltration of lymphoblastic cells in the BM from mouse #1376. (Right panel): Myeloblasts from mouse #1376 showed the expression of CD34 and c-Kit. C-lit was also expressed in myeloblasts from mouse #1348, whereas no expression of CD34 or C-Kit was observed in the BM cells from mouse #1436. **d** Cell surface and intracellular markers expressed by leukemic cells in the liver from mouse #1364 (ITD/ITD; *p53*^+/-^, T-ALL mouse). Intra CD3: intracellular CD3; intra CD19: intracellular CD19.

**Fig. S7 Development of solid tumor and lymphoma in *p53*^−/−^ mice.** **a** and **b** Histology showing the development of rhabdomyosarcoma in mouse #1345 (**a**, ×100; **b**, ×200). **c** and **d** Development of lymphoma in mouse #1317. **c** histology section of the thymus, ×200) **d** Cytospin of the thymus demonstrating lymphoblastic cells (×1000).

**Fig. S8 Analysis of ITD/ITD; *p53* KO mice with acute leukemia.** Analysis of the blood, bone marrow, spleen, liver, and lymph node of moribund ITD/ITD; *p53* KO mice demonstrated pathological features of acute leukemia. The recipient mice with AML showed marked hepatosplenomegaly (**a** and **b**), leukocytosis, reduced platelet count, and anemia (some cases) in the peripheral blood (**d**–**f**). Each dot represents one mouse.

**a**: ITD/ITD = 8, ITD/ITD; *p53*^+/−^ = 44

(**b** and **c**): ITD/ITD = 16, *p53*^+/−^ = 28, ITD/ITD; *p53*^+/−^ = 69

(**d**–**f**): ITD/ITD = 13, *p53*^+/−^ = 24, ITD/ITD; *p53*^+/−^ = 64


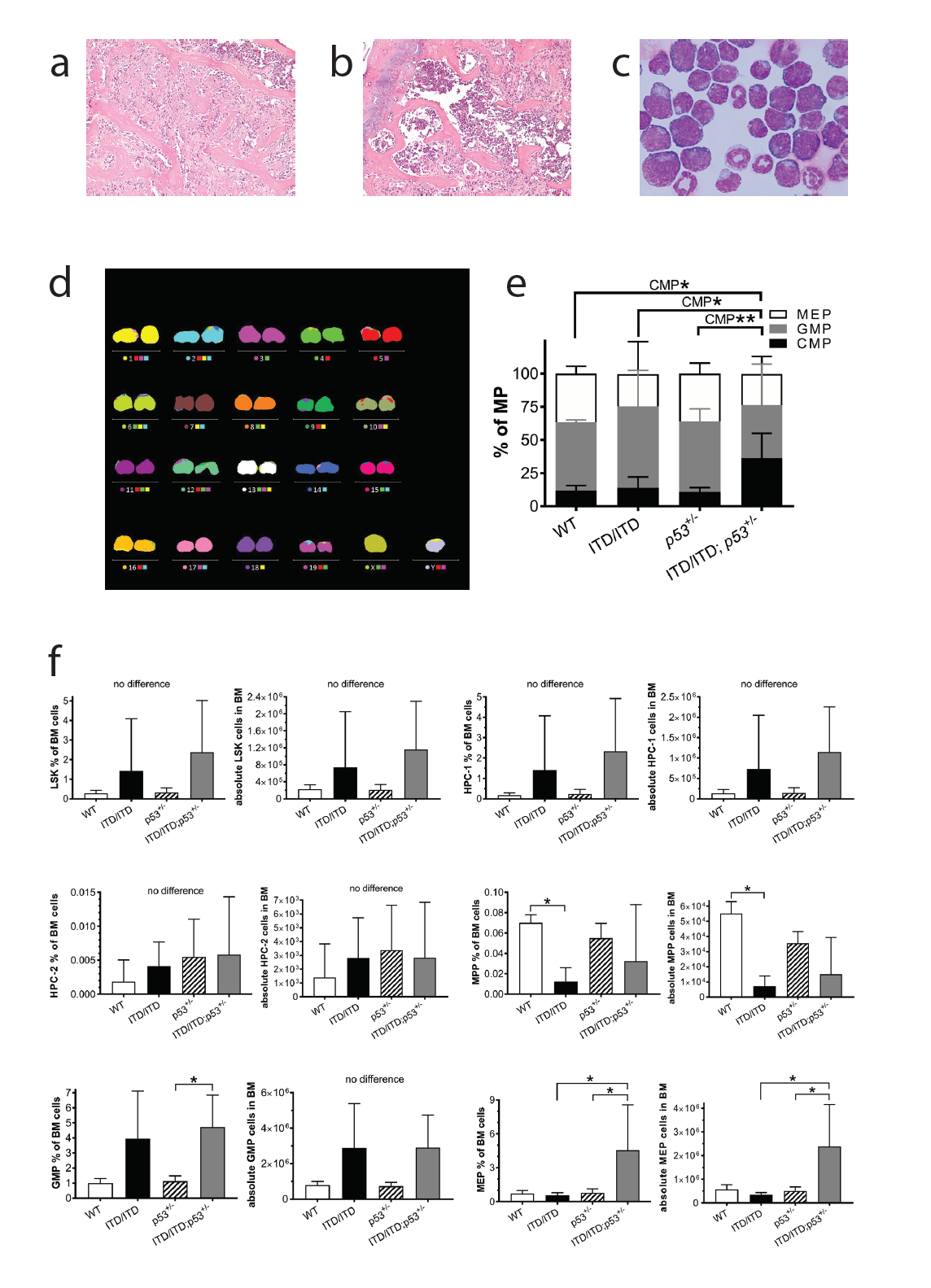


**Fig. S9 Leukemia development in ITD/ITD; *p53*^+/−^ mice. a–d** The development of AML and osteosarcoma in mouse #1350. **a** and **b** Histology showing osteosarcoma in BM. **c** Cytospin analysis showing myeloblasts in BM from mouse #1350. **d** mFISH analysis showing a normal karyotype for BM cells from mouse #1350. **e** Relative frequencies of CMP, GMP, and MEP cells in the MP fraction in ITD/ITD; *p53*^+/−^ mice compared with other groups. FLT3ITD/ITDP53+/- AML mice had a significant higher frequency of myeloid progenitors in BM than WT, FLT3ITD/ITD mice with MPN, and P53+/- mice, due to the expansion of common myeloid progenitors (CMP). The data shown in graphs represent the mean ± SD. * p < 0.05, ** p < 0.01. MP: myeloid progenitors (Lin-IL-7Rα-c-kit+Sca1-). WT (wild-type): n = 3 mice; ITD/ITD: n = 6; *p53*^+/−^: n = 6; ITD/ITD; *p53*^+/−^: n = 6. **f** Percentages and absolute cell number of LSK, HPC-1, HPC-2, MPP, GMP, and MEP cells in the BM. These cells in the ITD/ITD; p53+/- group were not significantly increased compared with that in WT mice. LSK: stem progenitors (Lin^-^c-kit^+^Sca-1^+^); HPC-1 (Lin^−^c-kit^+^Sca-1^+^CD48^+^CD150^-^) [25]; HPC-2 (Lin^−^c-kit^+^Sca-1^+^CD48^+^CD150^+^): MPP: multi-potent progenitors (Lin^−^c-kit^+^Sca-1^+^CD48^-^CD150^-^); MEP: megakaryocyte erythrocyte progenitors (Lin-IL-7Rα-c-Kit+Sca-1−CD34-FcgR−); GMP: granulocyte monocyte progenitor (Lin-IL-7Rα-c-Kit+Sca-1−CD34+FcgR+). WT (wild-type): n=3 mice; ITD/ITD: n=6; p53^+/-^: n=6; ITD/ITD; p53^+/-^: n=6.

**Fig. S10 Secondary transplantation of leukemic cells from ITD/ITD; *p53*^+/−^ mice with AML or T-ALL.** **a** Secondary recipient of ITD/ITD; *p53*^+/−^ AML cells developed leukemia that fully resembled the leukemic phenotype of the donor mouse #1376. Cytology and histology showing typical myeloblasts in mouse #1554. Immunophenotypic analyses of leukemic cells; the relative frequencies of CMP, GMP, and MEP cells in the MP compartment of BM from the secondary recipient (mouse #1554), comparable with the donor mouse #1376 (Figure 3). **b** Secondary recipients of ITD/ITD; *p53*^+/−^ T-ALL cells developed leukemia that fully recapitulated the disease of donor mouse #1346. Cytology and histology demonstrating the infiltration of lymphoblastic cells in different organs. Immunophenotype of leukemic cells from the secondary recipient mouse #1559, showing CD4/CD8 double-positive cells.


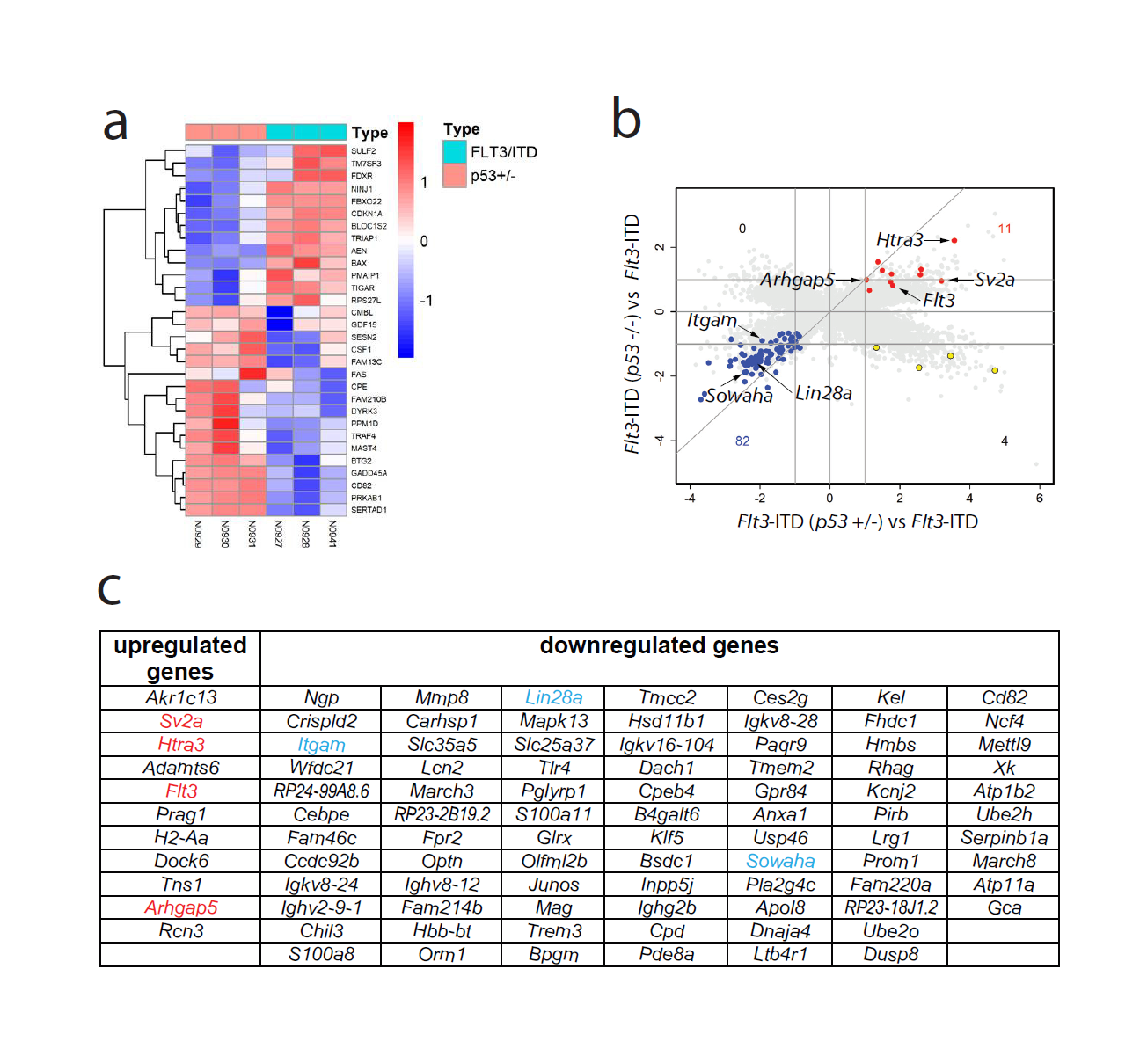


**Fig. S11 RNA-Seq analysis.** a Heatmap and hierarchical clustering of differentially expressed p53 target genes [26]. Values are normalized with log2. The right longitudinal axis indicates the names of differentially expressed genes. The left longitudinal axis represents the clustering information of differentially expressed genes. Red and blue denote upregulated and downregulated genes respectively. The expression of 13 *p53* target genes in ITD/ITD mice was higher than that of *p53*^+/-^ mice (P<0.05), and the expression of 17 *p53* target genes in ITD/ITD mice including *p21* (*CDKN1A*) was lower than that of *p53*^+/-^ mice (P<0.05). There was no significant difference in the expression of MDM2/MDMX between the two groups of mice. **b** “Four-way plot” and **c** showing 11 and 82 genes that were upregulated and downregulated, respectively. These genes were found during the analysis of two data sets (ITD/ITD; *p53*^+/−^ vs. ITD/ITD and ITD/ITD; *p53*^−/−^ vs. ITD/ITD). The seven selected genes for TaqMan assays were marked in red and blue. For Generation of „Four way plot“, the „Four way plot“ was generated as previously described [27].

Moreover, RNA-Seq analysis did not reveal the upregulation of *Otc* or *Arg1* by *FLT3*-ITD and *p53* KO (in contrast to *p53* KO solid tumor cells [28]), suggesting that the dysregulation of ammonia metabolism is unlikely to be involved in the development of AML in our model.

**Fig. S12** **Development of an efficient therapy for ITD/ITD; *p53* KO leukemia.** **a** Freshly isolated leukemic cells from mice with myeloid leukemia were sensitive to carfilzomib in a dose-dependent manner. Carfilzomib plus crenolanib showed additive cytotoxic effects against both ITD/ITD; *p53*^+/−^ T-ALL cells (n = 3 and 9 for ITD/ITD and ITD/ITD; *p53*^+/−^, respectively) and BM cells of AML mice (n = 2 and 3 for ITD/ITD and ITD/ITD; *p53*^+/−^, respectively). The combined killing effects in ITD/ITD; *p53*^+/−^ were comparable to those in ITD/ITD leukemic cells. The combination of carfilzomib and midostaurin also demonstrated additive killing effects against both ITD/ITD; *p53*^+/−^ T-ALL cells, and BM cells of AML mice (data not shown). Differentiation was not induced by combined therapy (data not shown). **b** Synergistic effects of combined therapies in the leukemic cell line from mouse #1358. All combined therapies were superior to single therapies. The results presented are the mean ± SD of at least three independent experiments. * p < 0.05, ** p < 0.01, *** p < 0.001.

**Fig. S13 Phosphorylation of AXL.** **a** Antibody array showing the phosphorylation of AXL in 32D cells expressing *AXL* and in HT1080 cells. WT 32D cells were used as the control. Hybridization signals at the corners served as positive controls. For these analyses, 32D cells were transduced with a lentiviral vector expressing human *AXL*. **b** Western blot analysis confirmed the phosphorylation of AXL in 32D *AXL* and HT1080 cells. The specificity and sensitivity of the array kit have been documented by reports from other groups [8,29].

**Table S1 mFISH analyses for each individual ITD/ITD; *p53* knockout leukemic mouse.**

| **Genotype** | **Mouse** | | **Karyotype**  **[number of**  **metaphases]** | **Tissue** |
| --- | --- | --- | --- | --- |
|  | | #1331  (CMML) | 40, XY [10] | BM |
|  | | #1340  (T-ALL/AML) | 40, XY [10] | BM |
|  | | #1343  (T-ALL/AML) | 40, XX [11]  40, XX [12] | BM  spleen |
|  | | #1348  (T-Lymphoblastic lymphoma/AML) | 40, XY [11] | spleen |
|  | | #1349  (AML) | 40, XX [10]  40, XX [10] | BM  spleen |
| ITD/ITD; p53^+/-^ | | #1350  (AML/Osteosarcoma) | 40, XY [10] | BM |
|  | | #1353  (T-Lymphoblastic lymphoma/AML) | 40, XX [12] | BM |
|  | | #1354  (T-ALL/AML) | 40, XX [12] | spleen |
|  | | #1356  (AML) | 40, XX [15] | spleen |
|  | | #1357  (CMML) | 40, XX [12] | spleen |
| ITD/ITD; p53^-/-^ | | #1344  (AML) | 40, XY [12] | BM |
|  | | #1352  (T-ALL/AML) | 40, XX [11]  40, XX [8] | Spleen, thymus |

**Table S2 Limiting dilution transplantation of leukemic cells. Direct comparison between #1338 and #1358 cells.** The combination of ITD/ITD and *p53* KO even reduced the serial replanting activity *in vivo*.

| Transplanted cell number | Donor: #1358 cells  (ITD/ITD; *p53*^+/-^)  disease penetrance | Donor: #1338 cells  (ITD/ITD)  disease penetrance |
| --- | --- | --- |
| 1 | 0/3 | ND |
| 2 | 0/2 | 0/3 |
| 10 | 0/3 | 0/3 |
| 10^2^ | 0/3 | 1/3 |
| 10^3^ | 0/3 | 1/3 |
| 10^4^ | ½ | 3/3 |
| 10^5^ | 1/1 | 3/3 |
| 10^6^ | ND | 3/3 |

ND= not done

**Table S3 Gene ontology analysis identified pathways affected by the cooperation between ITD/ITD and *p53* KO in two pairwise comparisons (ITD/ITD; *p53*^+/−^ vs. ITD/ITD and ITD/ITD; *p53*^−/−^ vs. ITD/ITD).**

Pathways P value

GO:0006954~inflammatory response 0.0010

GO:0042592~homeostatic process 0.0016

GO:0061515~myeloid cell development 0.003

GO:0071621~granulocyte chemotaxis 0.009

GO:0050663~cytokine secretion 0.006

GO:0048821~erythrocyte development 0.01

GO:0045620~negative regulation of lymphocyte differentiation 0.01

GO:0001816~cytokine production 0.01

GO:0097530~granulocyte migration 0.01

GO:0030097~hemopoiesis 0.01

GO:0002262~myeloid cell homeostasis 0.03

GO:0030099~myeloid cell differentiation 0.02

**Table S4 Plasma concentrations of R428 and carfilzomib**

|  | **Plasma concentration (nM) in treated animals (mean)** | **Number of animals tested** |
| --- | --- | --- |
| **R428** | | |
| 2h after treatment | 4885-7600 (5759) | 5 |
| 6h | 4065-6865 (5555) | 3 |
| 24h | 1905, 3545 (2725) | 2 |
| **Carfilzomib** | | |
| 0.5 min after treatment | 1050-1140 (1087) | 3 |
| 2 min | 298, 810 (554) | 2 |
| 5 min | 85-414 (227) | 3 |
| 15 min | 0, 191 (96) | 2 |

R428 exhibited a long plasma half- life as reported (about 4 hours at 25 mg/kg in a study [9]), while plasma half- life of carfilzomib was very short, in consistent with a report [30].

**References**

1. Schlenk RF, Kayser S, Bullinger L, Kobbe G, Casper J, Ringhoffer M, et al. . Differential impact of allelic ratio and insertion site in FLT3-ITD-positive AML with respect to allogeneic transplantation. Blood. 2014;124:3441-3449.

2. Quentmeier H, Reinhardt J, Zaborski M, Drexler HG. FLT3 mutations in acute myeloid leukemia cell lines. Leukemia. 2003;17:120-124.

3. Li L, Bailey E, Greenblatt S, Huso D, Small D. Loss of the wild-type allele contributes to myeloid expansion and disease aggressiveness in FLT3/ITD knockin mice. Blood. 2011;118:4935-4945.

4. Lange K, Gadzicki D, Schlegelberger B, Gohring G. Recurrent involvement of heterochromatic regions in multiple myeloma-a multicolor FISH study. Leuk Res. 2010;34:1002-1006.

5. Zhao L, Melenhorst JJ, Alemu L, Kirby M, Anderson S, Kench M, et al. . KIT with D816 mutations cooperates with CBFB-MYH11 for leukemogenesis in mice. Blood. 2012;119:1511-1521.

6. Li Z, Dullmann J, Schiedlmeier B, Schmidt M, von Kalle C, Meyer J, et al. . Murine leukemia induced by retroviral gene marking. Science. 2002;296:497.

7. Li Z, Beutel G, Rhein M, Meyer J, Koenecke C, Neumann T, et al. . High-affinity neurotrophin receptors and ligands promote leukemogenesis. Blood. 2009;113:2028-2037.

8. Stommel JM, Kimmelman AC, Ying H, Nabioullin R, Ponugoti AH, Wiedemeyer R, et al. . Coactivation of receptor tyrosine kinases affects the response of tumor cells to targeted therapies. Science. 2007;318:287-290.

9. Holland SJ, Pan A, Franci C, Hu Y, Chang B, Li W, et al. . R428, a selective small molecule inhibitor of Axl kinase, blocks tumor spread and prolongs survival in models of metastatic breast cancer. Cancer Res. 2010;70:1544-1554.

10. Chen S, Wang Q, Yu H, Capitano ML, Vemula S, Nabinger SC, et al. . Mutant p53 drives clonal hematopoiesis through modulating epigenetic pathway. Nat Commun. 2019;10:5649.

11. Nabinger SC, Chen S, Gao R, Yao C, Kobayashi M, Vemula S, et al. . Mutant p53 enhances leukemia-initiating cell self-renewal to promote leukemia development. Leukemia. 2019;33:1535-1539.

12. Chopra M, Bohlander SK. The cell of origin and the leukemia stem cell in acute myeloid leukemia. Genes Chromosomes Cancer. 2019;58:850-858.

13. Gollner S, Oellerich T, Agrawal-Singh S, Schenk T, Klein HU, Rohde C, et al. . Loss of the histone methyltransferase EZH2 induces resistance to multiple drugs in acute myeloid leukemia. Nat Med. 2017;23:69-78.

14. Jin Y, Nie D, Li J, Du X, Lu Y, Li Y, et al. . Gas6/AXL Signaling Regulates Self-Renewal of Chronic Myelogenous Leukemia Stem Cells by Stabilizing beta-Catenin. Clin Cancer Res. 2017;23:2842-2855.

15. Gay CM, Balaji K, Byers LA. Giving AXL the axe: targeting AXL in human malignancy. Br J Cancer. 2017;116:415-423.

16. Mupo A, Celani L, Dovey O, Cooper JL, Grove C, Rad R, et al. . A powerful molecular synergy between mutant Nucleophosmin and Flt3-ITD drives acute myeloid leukemia in mice. Leukemia. 2013;27:1917-1920.

17. Yang L, Rodriguez B, Mayle A, Park HJ, Lin X, Luo M, et al. . DNMT3A Loss Drives Enhancer Hypomethylation in FLT3-ITD-Associated Leukemias. Cancer Cell. 2016;29:922-934.

18. Meyer SE, Qin T, Muench DE, Masuda K, Venkatasubramanian M, Orr E, et al. . DNMT3A Haploinsufficiency Transforms FLT3ITD Myeloproliferative Disease into a Rapid, Spontaneous, and Fully Penetrant Acute Myeloid Leukemia. Cancer Discov. 2016;6:501-515.

19. Prokocimer M, Molchadsky A, Rotter V. Dysfunctional diversity of p53 proteins in adult acute myeloid leukemia: projections on diagnostic workup and therapy. Blood. 2017;130:699-712.

20. Zhao Z, Zuber J, Diaz-Flores E, Lintault L, Kogan SC, Shannon K, et al. . p53 loss promotes acute myeloid leukemia by enabling aberrant self-renewal. Genes Dev. 2010;24:1389-1402.

21. Armstrong SA, Mabon ME, Silverman LB, Li A, Gribben JG, Fox EA, et al. . FLT3 mutations in childhood acute lymphoblastic leukemia. Blood. 2004;103:3544-3546.

22. Network. CGAR. Genomic and epigenomic landscapes of adult de novo acute myeloid leukemia. N Engl J Med. 2013;368:2059-2074.

23. Gao Jea. Integrative Analysis of Complex Cancer Genomics and Clinical Profiles Using the cBioPortal. Sci Signal. 2013;6:pl1.

24. Cerami Eea. The cBio cancer genomics portal: an open platform for exploring multidimensional cancer genomics data. Cancer Discov. 2012;2:401-404.

25. Oguro H, Ding L, Morrison SJ. SLAM family markers resolve functionally distinct subpopulations of hematopoietic stem cells and multipotent progenitors. Cell Stem Cell. 2013;13:102-116.

26. Fischer M. Census and evaluation of p53 target genes. Oncogene. 2017;36:3943-3956.

27. Prajeeth CK, Dittrich-Breiholz O, Talbot SR, Robert PA, Huehn J, Stangel M. IFN-gamma Producing Th1 Cells Induce Different Transcriptional Profiles in Microglia and Astrocytes. Front Cell Neurosci. 2018;12:352.

28. Li L, Mao Y, Zhao L, Li L, Wu J, Zhao M, et al. . p53 regulation of ammonia metabolism through urea cycle controls polyamine biosynthesis. Nature. 2019;567:253-256.

29. Chandarlapaty S, Sawai A, Scaltriti M, Rodrik-Outmezguine V, Grbovic-Huezo O, Serra V, et al. . AKT inhibition relieves feedback suppression of receptor tyrosine kinase expression and activity. Cancer Cell. 2011;19:58-71.

30. Yang J, Wang Z, Fang Y, Jiang J, Zhao F, Wong H, et al. . Pharmacokinetics, pharmacodynamics, metabolism, distribution, and excretion of carfilzomib in rats. Drug Metab Dispos. 2011;39:1873-1882.
